# LC-MS-Based Plasma Metabolomics and Lipidomics Analyses for Differential Diagnosis of Bladder Cancer and Renal Cell Carcinoma

**DOI:** 10.3389/fonc.2020.00717

**Published:** 2020-05-15

**Authors:** Xiang Liu, Mingxin Zhang, Xiangming Cheng, Xiaoyan Liu, Haidan Sun, Zhengguang Guo, Jing Li, Xiaoyue Tang, Zhan Wang, Wei Sun, Yushi Zhang, Zhigang Ji

**Affiliations:** ^1^Institute of Basic Medical Sciences, School of Basic Medicine, Peking Union Medical College, Chinese Academy of Medical Sciences, Beijing, China; ^2^Department of Urology, Peking Union Medical College Hospital, Chinese Academy of Medical Science, Beijing, China; ^3^Department of Urology, The Affiliated Hospital of Qingdao University, Qingdao, China

**Keywords:** bladder cancer, renal cell carcinoma, metabolomics, lipidomics, biomarker

## Abstract

Bladder cancer (BC) and Renal cell carcinoma(RCC) are the two most frequent genitourinary cancers in China. In this study, a comprehensive liquid chromatography—mass spectrometry (LC-MS) based method, which utilizes both plasma metabolomics and lipidomics platform, has been carried out to discriminate the global plasma profiles of 64 patients with BC, 74 patients with RCC, and 141 healthy controls. Apparent separation was observed between cancer (BC and RCC) plasma samples and controls. The area under the receiving operator characteristic curve (AUC) was 0.985 and 0.993 by plasma metabolomics and lipidomics, respectively (external validation group: AUC was 0.944 and 0.976, respectively). Combined plasma metabolomics and lipidomics showed good predictive ability with an AUC of 1 (external validation group: AUC = 0.99). Then, separation was observed between the BC and RCC samples. The AUC was 0.862, 0.853 and 0.939, respectively, by plasma metabolomics, lipidomics and combined metabolomics and lipidomics (external validation group: AUC was 0.802, 0.898, and 0.942, respectively). Furthermore, we also found eight metabolites that showed good predictive ability for BC, RCC and control discrimination. This study indicated that plasma metabolomics and lipidomics may be effective for BC, RCC and control discrimination, and combined plasma metabolomics and lipidomics showed better predictive performance. This study would provide a reference for BC and RCC biomarker discovery, not only for early detection and screening, but also for differential diagnosis.

## Introduction

Bladder cancer (BC) and Renal cell carcinoma(RCC) are, respectively, the second and third most common genitourinary cancers in Europe and North America, and the first two most commonly occurring genitourinary cancers in China (1). Currently, cystoscopy and cytology are the standards for initial BC diagnosis and recurrence, but they have some limitations. Cystoscopy may fail to visualize certain areas within the bladder, and may also fail to detect some cancers, particularly cases of carcinoma *in situ* (2). Cytology has high specificity and selectivity for high-grade tumors, but fails to provide a strong predictive value for low-grade tumors (3). With regard to RCC, computed tomography, magnetic resonance imaging, and positron emission tomography are commonly used diagnostic techniques (4). However, even with the combination of these three techniques, it remains difficult to detect early tumors because of their small size (5). Therefore, developing convenient and novel techniques for early detection of BC and RCC with high sensitivity and specificity is urgently required. There are increasing numbers of studies evaluating the use of metabolomic analyses in the diagnosis of a number of pathologies (6–8) and in the elucidation of the clinical pathogenesis of various diseases (9, 10). Lipidomics is an emerging independent branch of metabolomics (11), and lipid metabolism dysfunction has been found to be associated with the pathogenesis of many diseases, such as ovarian cancer (12), prostate cancer (13), and breast cancer (14), among others.

Metabolomics has also been used to study BC and RCC, especially to identify biomarkers in urine and serum (15–23). In 2014, Jin et al. (23) applied LC-MS to profile urinary metabolites of 138 patients with BC and 121 control subjects. The study identified 12 putative markers that were involved in glycolysis and beta-oxidation; Wittmann et al. (19) applied LC-MS to profile urinary metabolites of 66 BC and 266 non-BC subjects. They suggested that metabolites (palmitoyl sphingomyelin, phosphocholine, and arachidonate) related to lipid metabolism may be potential BC markers. In 2016, Zhou et al. (20) developed a plasma pseudotargeted method based on GC-MS SIM and found metabolites involved in the PPP, nucleic acid, and fatty acid biosynthesis were disordered in BC patients. For RCC research, in 2011, Kim et al. (16) analyzed urine metabolomics of 29 kidney cancer patients and 33 control patients and identified 13 significant differentially expressed metabolites (hexanoylglycine, 4-hydroxybenzoate, gentisate, etc) that involved in amino acid metabolism and fatty acid beta-oxidation metabolism. In 2017, Falegan et al. (18) applied an NMR and GC-MS platform to perform urine and serum metabolomics for 40 RCC patients and 13 benign patients. The results showed alterations in levels of glycolytic and tricarboxylic acid (TCA) cycle intermediates in RCC relative to benign masses. In addition, Lin et al. (5) have utilized both RPLC-MS and HILIC-MS to discriminate the global serum profiles of BC, RCC, and non-cancer controls. The study identified some cancer-specific potential biomarkers for BC and RCC, and they also found acetylphenylalanine, methyl hippuric acid, PC(40:7) and PC(40:6) were common differential biomarkers for both BC and RCC. As described, these studies showed the same changes of pathways, including glycolysis, amino acid metabolism and fatty acid metabolism in BC and RCC patients, but there is less consistency in identified metabolites in these studies (**Table 5**).

As mentioned above, previous studies have identified some potential disease biomarkers in urine and serum for BC or RCC diagnosis, but some issues remain to be addressed. First, most of these studies focus on one kind of cancer. However, in clinic there is great interest in the possibility of distinguishing different types of cancer based on metabolomics and to acquire deeper insight into the tumor biology and cancer type-specific biomarker discovery (5, 24, 25). Up to now, only one study worked on above issue. Lin et al. (5) utilized serum metabolomics to discriminate the global serum profiles of BC, RCC, and non-cancer controls. The results indicated that serum metabolic profiling could be used for BC or RCC diagnosis. They also identified some metabolites that were common differential biomarkers for both BC and RCC. Lin et al. study provided very useful metabolomic clues for BC and RCC common biomarker discovery, but their conclusions and results needed more work to be proved. In addition, it remains to explore whether serum metabolomics could be used for differential diagnosis of genitourinary cancer (16, 18, 19, 23). Second, to our knowledge, urinary metabolomics has been extensively investigated for BC and RCC biomarker discovery (15, 18, 19, 26–28), but there are few studies on blood metabolomics and lipidomics for BC biomarker discovery. Blood has fewer intra- and inter-individual variations, and it is less susceptible to dietary changes than urine (29). Moreover, blood is rich in lipids, which plays an essential role in many biological processes (30). Lipidomics is proposed as a viable method to monitor the prognosis, diagnosis, and treatment of cancer and acts as a new method of cancer biomarker discovery (31). Therefore, the combination of metabolomics and lipidomics may be a significant platform for BC and RCC biomarker discovery.

In this study, we tried to explore potential biomarkers for BC and RCC, which can not only screen BC and/or RCC before subjective symptom in non-metastatic stage of cancer, but also provide differential diagnostic clues for BC or RCC in the clinical stage, so that the proper following tests (cystoscopy or computed tomography) can be used. Plasma metabolomics and lipidomics were utilized, first to explore potential biomarkers between cancer (BC and RCC) and non-cancer. Then, differential metabolites were explored between BC and RCC to find cancer-specific biomarker for differential diagnosis. Furthermore, we also explored common differential metabolites among BC, RCC, and control groups to find whether it is a panel of metabolites biomarker could be as potential biomarker for discrimination of BC, RCC, and control. Our study will provide a reference for BC and RCC biomarker discovery, not only for early detection and screening, but also for differential diagnosis.

## Materials and Methods

### Sample Collection and Preparation

The consent procedure and the research protocol for this study were approved by the Institutional Review Board of the Institute of Basic Medical Sciences, Chinese Academy of Medical Sciences (Project NO: 047-2019). And all participants provided informed consent and took a series of physical examinations and laboratory tests before participating in this study, including blood pressure, body mass index (BMI), fasting blood glucose (FBG), total cholesterol (TC), triglyceride (TG) etc. Finally, a total of 141 participants aged 27–74 years with health standard were recruited in this study. Meanwhile, the BC and RCC patients also took above tests, and only the patients with normal results were recruited.

The plasma samples from 64 bladder cancer (BC) patients, 74 Renal cell carcinoma(RCC) patients and 141 healthy controls were collected from Peking Union Hospital (Table 1, the detailed clinical information was shown in Table S1). All the plasma samples in our study were collected before any treatments. The plasma samples were collected in the morning from 07:00 a.m.−09:00 a.m. after an overnight fast to eliminate dietary disturbances. After collected, all plasma samples were separated following centrifugation at 1,024 g for 10 min at 4°C and were stored at −80°C.

**Table 1:** Demographics of cancer (BC and RCC) patients and healthy controls.

**Sample group**	**Discovery group**			**Validation group**		
	**Healthy controls**	**BC patients**	**RCC patients**	**Healthy controls**	**BC patients**	**RCC patients**
No. plasma samples	95	42	53	46	22	21
Mean age ± SD	59.25 ± 11.19	64.21 ± 14.18	56.96 ± 15.09	61.32 ± 9.43	62.59 ± 12.77	53.66 ± 12.35
No. Males	65	31	36	30	14	16
No. Females	30	11	17	16	8	5

### Sample Preparation

For plasma metabolomics, 50 μL of sample were mixed with 150 μL of H_2_O by vortexed for 30 s to dilute the sample, then acetonitrile (400 μl) was added into each sample (200 μl), the mixture was vortexed for 1 min. The mixture was allowed to stand for 30 min at −20°C and was centrifuged at 14,000 × g for 10 min. The supernatant was dried under vacuum and then reconstituted with 100 μL of 2% acetonitrile. For plasma lipidomics, 200 μL plasma samples were precipitated by the addition of 600 μL of isopropanol (IPA) precooled to −20°C. Samples were stored for 2 h at −20°C to improve protein precipitation and then centrifuged at 14,000 × g for 20 min. The supernatant was dried under vacuum and then reconstituted with 100 μL of 50% IPA. The quality control (QC) (32) sample was a pooled sample prepared by mixing aliquots of two hundred samples across different groups. And the two hundred samples were randomly selected from BC, RCC and control groups.

### LC-MS Analysis

Ultra-performance LC-MS analyses of samples were conducted using a Waters ACQUITY H-class LC system coupled with an LTQ-Orbitrap Velos mass spectrometer (Thermo Fisher Scientific, MA, USA). An HSS C18 column (3.0 × 100 mm, 1.7 μm) (Waters, Milford, MA, USA) was used for reversed phase separation. Plasma metabolites were separated with an 18 min gradient at a flow rate of 0.5 mL/min. Mobile phase A was 0.1% formic acid in H_2_O and mobile phase B was acetonitrile. The gradient was set as follows: 0–1 min, 2% solvent B; 1–3 min, 2–55% solvent B; 3–8 min, 55–100% solvent B; 8–13 min, 100% solvent B; 13–13.1 min, 100–2% solvent B; 13.1–18 min, 2% solvent B. The column temperature was set as 50°C. Plasma lipids were separated with a 23 min gradient at a flow rate of 0.4 mL/min. Mobile phase A was 10 mM ammonium acetate in acetonitrile (4:6) and mobile phase B was 10 mM ammonium acetate in isopropanol/acetonitrile (9:1). The gradient was set as follows: 0 min, 40% solvent B; 0–2 min, 40–43% solvent B; 2–8 min, 43–85% solvent B; 8–15 min, 85–99% solvent B; 15–18 min, 99% solvent B; 18–18.1 min, 99–40% solvent B; 18.1–23 min, 40% solvent B. The column temperature was set as 55°C.

The mass spectrometer was operated in positive ion mode using the m/z range 100–1,000 m/z at a resolution of 60 K. Automatic gain control (AGC) target was 1 × 10^6^ and maximum injection time (IT) was 100 ms. Subsequently differential metabolites identification was performed by UPLC targeted-MS/MS analyses of QC sample. It acquired at a resolution of 15 K with AGC target of 5 × 10^5^, maximum IT of 50 ms, and isolation window of 3 m/z. Collision energy was optimized as 20, 40, 60 for each target with higher-energy collisional dissociation (HCD) fragmentation.

### Data Processing

Raw data files (Figure S6) were processed by the Progenesis QI 2.2 (Waters, Milford, MA, USA) software (33). The detailed workflow for QI data processing and metabolites identification was given in Supplementary Methods. Further data pre-processing including missing value estimation, Log transformation and Pareto scaling were performed to make features more comparable using MetaboAnalyst 4.0 (34) (http://www.metaboanalyst.ca). Pattern recognition analysis (principal component analysis, PCA; orthogonal partial least squares discriminant analysis, OPLS-DA) was carried out using SIMCA 14.0 software (Umetrics, Sweden). The differential variables were selected according to three conditions: (1) adjusted *P* < 0.05; 2) Fold change between two groups >1.5; 3) VIP value obtained from OPLS-DA > 1.

### Metabolite Annotation and Pathway Analysis

Significantly differential metabolites were further determined from the exact mass composition, from the goodness of the isotopic fit for the predicted molecular formula and from MS/MS fragmentation matching with databases (HMDB (35), LIPID MAPS, METLIN, and mzCloud), using Progenesis QI 2.2 (Waters, Milford, MA, USA). In addition, homocysteine thiolactone, hypoxanthine, 4-Ethylphenol, L-Octanoylcarnitine and acetylcysteine were confirmed by standard compounds (Figure S7). Metabolic pathways were analyzed using Mummichog (36) and MetaboAnalyst 4.0 (34). Identified differential metabolites were subjected to MetaboAnalyst 4.0 to perform exploratory ROC analysis. Random Forest algorithms were used for ROC curve construction. Detailed methods were listed in the Supplemental Methods.

## Results

**Figure 1:**
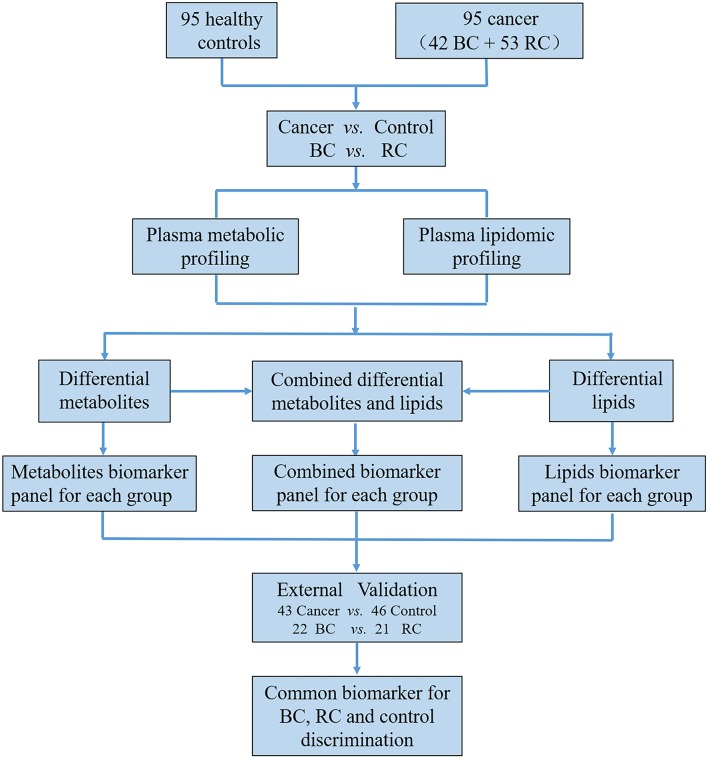
The workflow of this study.

The workflow of this study is shown in Figure 1 total of 279 subjects were enrolled in our study, with 141 volunteers with a normal clinically healthy index, 64 patients who were clinically diagnosed with bladder cancer and 74 patients who were clinically diagnosed with Renal cell carcinoma. First, LC-MS based plasma metabolomics and lipidomics were performed based on 95 healthy controls, 42 patients with BC and 53 patients with RCC. Differential metabolites were found through a critical selection criterion. Potential biomarkers for cancer vs. control and BC vs. RCC were explored and discovered tentatively. Moreover, the identified differential metabolites were also combined for better predictive ability. Then, the potential biomarkers were further externally validated using an independent batch of 22 BC, 21 RCC and 46 control samples. Furthermore, common differential metabolites were explored for BC, RCC, and control discrimination.

### Quality Control

This large cohort of samples was analyzed randomly in a single batch. QC is important in large-scale metabolomics studies to ensure stable system performance and to limit experimental bias. A QC standard was prepared as a pooled mixture of aliquots from representative plasma samples in each group. For plasma metabolomics and lipidomics analysis, the QC sample was injected 5 times before the analytical run and was frequently injected once every ten samples throughout the analytical run to monitor instrument stability. Metabolomics technical reproducibility was assessed by analyzing the QC sample variations with time. The injections showed a stable condition with small variation (< ± 2SD) in plasma metabolomics and lipidomics (Figures S1A,B). Tight clustering of QC samples (Figures S1C,D) further demonstrated the quality of the QC data and the essential repeatability and stability throughout the analytical run.

### Distinction Cancer (BC and RCC) From Control by Plasma Metabolomics and Lipidomics

#### Distinction Cancer (BC and RCC) From Control by Plasma Metabolomics

**Figure 2:**
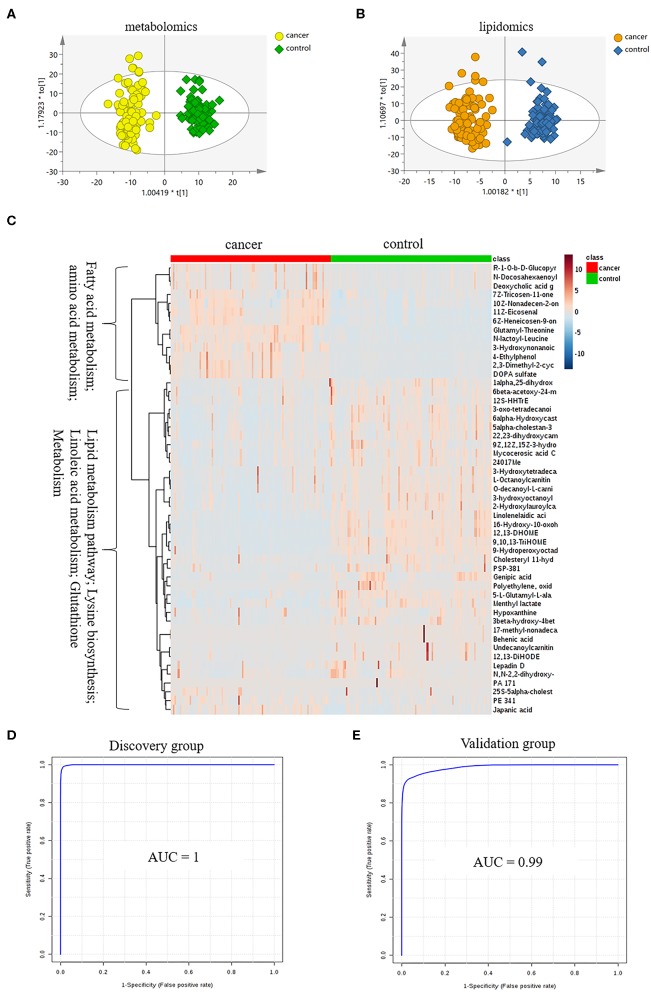
Analysis of plasma metabolomics and lipidomics of 95 cancer samples (42 BC and 53 RCC) and 95 healthy control samples. **(A)** Score plot of OPLS-DA based on plasma metabolic profiling of cancer and control. **(B)** Score plot of OPLS-DA based on plasma lipidomic profiling of cancer and control. **(C)** Relative intensity of differential metabolites in cancer and control. **(D)** ROC plot with discovery group for distinction of cancer and control based on combined metabolites panel of 9,10,13-TriHOME, 11Z-Eicosenal, 12,13-DHOME, 6Z-Heneicosen-9-one, linolenelaidic acid, behenic acid and 16-Hydroxy-10-oxohexadecanoic acid. **(E)** ROC plot with external validation group for distinction of cancer and control based on combined metabolites panel.

LC-MS-based plasma metabolomics from cancer and control patients yielded 2,432 spectral features after removal of missing values and quality control. To select potential biomarkers for distinguishing cancer (BC and RCC) from control patients, multivariate statistical analysis models were applied. Apparent differences between the metabolic profiles of cancer and control subjects was observed from the PCA score plot (R2X = 0.624, Q2 = 0.416; Figure S2A). The OPLS-DA model achieved better separation (R2X = 0.263, R2Y = 0.953, Q2 = 0.931; Figure 2A; Table S2). Permutation tests were carried out to confirm the stability and robustness of the supervised models presented in this study (Figure S2B). Differential metabolites were assigned based on VIP value >1, *p* < 0.05 and FC >1.5. Pathway enrichment analysis using Mummichog showed significant enrichment (*p* < 0.05) of several pathways related to tyrosine metabolism, linoleate metabolism, porphyrin metabolism, fructose, and mannose metabolism, and phosphatidylinositol phosphate metabolism, among others (Figure S2C), in cancer compared with that in the healthy controls.

Further, significantly differential features obtained from “mummichog” and OPLS-DA predictions were submitted to MS/MS fragmentation and Progenesis QI identification. Overall, 25 significantly differential metabolites were identified as shown in Table S3. The diagnostic accuracy of identified differential metabolites for cancer (BC and RCC) from control samples was evaluated. A total of 22 metabolites had a good diagnostic value with the AUC above 0.8 (37) (Table S4). Combined biomarkers are more valuable for diagnosing disease progression than just one biomarker (23). Multivariate ROC curve-based exploratory analysis was tried to achieve a better predictive model (https://www.metaboanalyst.ca/faces/upload/RocUploadView.xhtml) using these differential metabolites. A panel consisting of 9,10,13-TriHOME, 12,13-DHOME and linolenelaidic acid showed the best predictive ability with a ROC area of 0.985 for the testing dataset (Figure S2D) and 0.944 for the external validation dataset (Figure S2E).

#### Distinction Cancer (BC and RCC) From Control by Plasma Lipidomics

LC-MS-based plasma lipidomics from cancer and control samples was analyzed using similar multiple statistic methods as above. In total, 1421 spectral features were retained after quality control. PCA analysis showed apparent discrimination of cancer and control samples (R2X = 0.682, Q2 = 0.406; Figure S3A). Further, the OPLS-DA model achieved significant separation (R2X = 0.296, R2Y = 0.949, Q2 = 0.924; Figure 2B). Permutation tests showed stability and robustness of the supervised models (Figure S3B). Pathway enrichment analysis using Mummichog showed significant enrichment pathways related to the carnitine shuttle, the urea cycle/amino group metabolism, and fatty acid metabolism, among others (Figure S3C), in cancer compared with control samples. Overall, 26 significantly differential lipids were identified as shown in Table S5, and a total of 20 lipids had potential useful diagnostic values with the AUC above 0.7 (Table S6). A panel consisting of 11Z-Eicosenal, 6Z-Heneicosen-9-one, behenic acid and 7Z-Tricosen-11-one showed the best predictive ability with ROC area of 0.993 for the testing dataset (Figure S3D) and 0.976 for the external validation dataset (Figure S3E).

#### Distinction Cancer (BC and RCC) From Control by Combination of Plasma Metabolomics and Lipidomics

Combining the results of identified differential metabolites, the relative intensity was plotted as a heatmap in Figure 2C. It showed that the metabolites involved in amino acid metabolism and fatty acid metabolism were up-regulated in cancer patients, including dipeptides, bile acid metabolites, and some fatty acyls (FAs). While the down-regulated metabolites included some carnitines (3-hydroxyoctanoyl carnitine, L-Octanoylcarnitine, 2-Hydroxylauroylcarnitine, O-decanoyl-L-carnitine, Undecanoylcarnitine), glycerophospholipids (GPs), sphingolipids (SPs), and sterol lipids (STs). Multivariate ROC curve-based exploratory analysis was tried to achieve a better predictive model using these combined differential metabolites. A panel consisting of 9,10,13-TriHOME, 11Z-Eicosenal, 12,13-DHOME, 6Z-Heneicosen-9-one, linolenelaidic acid, behenic acid, and 16-Hydroxy-10-oxohexadecanoic acid (Table 2) showed the best predictive ability with ROC area of 1 for the testing dataset (Figure 2D) and 0.99 for the external validation dataset (Figure 2E).

**Table 2:** Differential metabolites for distinction of cancer (BC and RCC) and control.

**Features**	**Metabolites ID**	**Description**	**Score**	***p*-value**	**Fold change (cancer/HC)**	**AUC**
5.81_269.2104m/z	HMDB41287	16-Hydroxy-10-oxohexadecanoic acid^**a**^	45.3	2.97E-29	0.0988	0.9948
5.60_331.2470m/z	HMDB04710	9,10,13-TriHOME^**a**^	42.3	1.09E-28	0.1001	0.9853
6.60_314.2448n	HMDB04705	12,13-DHOME^**a**^	42.7	6.90E-27	0.1859	0.9675
6.59_279.2309m/z	HMDB30964	Linolenelaidic acid^**a**^	38.1	5.15E-23	0.4242	0.9313
9.09_311.3170n	LMFA06000248	11Z-Eicosenal^**b**^	50.9	5.89E-27	2.2601	0.9788
9.24_325.3325n	LMFA12000215	6Z-Heneicosen-9-one^**b**^	41.5	1.27E-24	2.2569	0.9535
8.19_358.3658m/z	LMFA01020019	Behenic acid^**b**^	48.9	2.53E-17	0.161	0.8726
9.71_354.3710m/z	LMFA12000222	7Z-Tricosen-11-one^**b**^	48.7	7.95E-12	1.6531	0.8049

a *Metabolites identified by the chemical structure analysis matching with The Human Metabolome Database*.

b *Metabolites identified by the chemical structure analysis matching with LIPID MAPS*.

### Distinction BC and RCC by Plasma Metabolomics and Lipidomics

BC and RCC are the first two most frequent genitourinary cancers in China. The above analysis explored potential differential metabolites to discriminate cancer (BC and RCC) from control samples, and the feasibility of using plasma metabolomics and lipidomics to discover potential biomarkers for differential diagnosis of the two types of cancer was evaluated.

#### Distinction BC and RCC by Plasma Metabolomics

**Figure 3:**
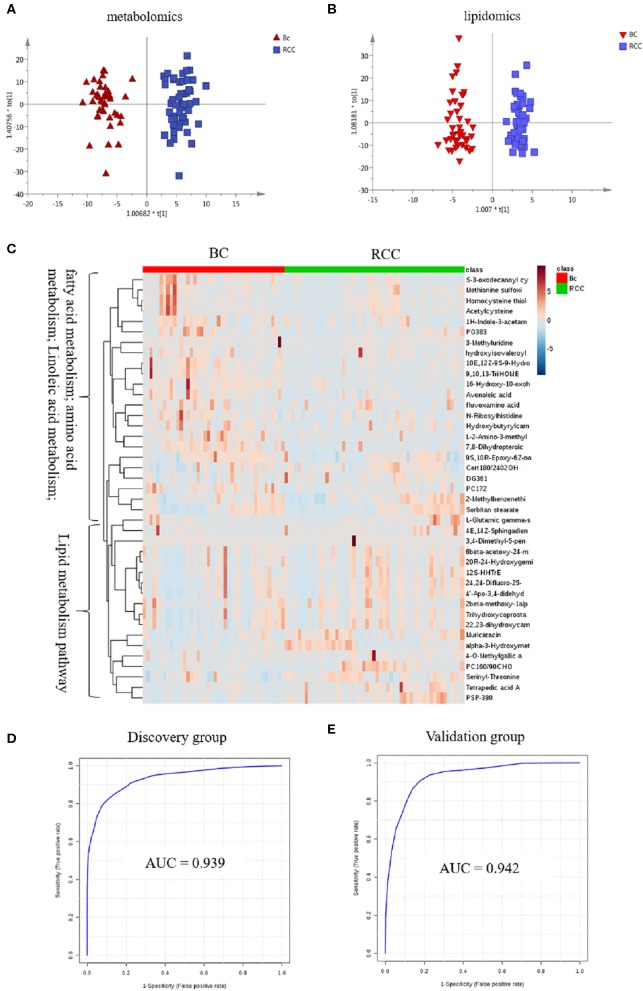
Analysis of plasma metabolomics and lipidomics between 42 BC and 53 RCC. **(A)** Score plot of OPLS-DA based on plasma metabolic profiling of BC and RCC. **(B)** Score plot of OPLS-DA based on plasma lipidomic profiling of BC and RCC. **(C)** Relative intensity of differential metabolites in BC and RCC. **(D)** ROC plot with discovery group for distinction of BC and RCC based on combined metabolites panel of 7,8-Dihydropteroic acid, PS(P-38:0), 9,10,13-TriHOME, Avenoleic acid, 3,4-Dimethyl-5-pentyl-2-furanundecanoic acid and 4E,14Z-Sphingadiene. **(E)** ROC plot with external validation group for discrimination of BC and RCC based on combined metabolites panel.

Herein, using similar multiple statistic methods as above, metabolic profiling differentiation was explored between BC and RCC plasma samples. First, the metabolic profiles of BC and RCC subjects showed separation trend to some extent from the PCA score plot (R2X = 0.557, Q2 = 0.324; Figure S4A). The OPLS-DA model achieved better separation (R2X = 0.322, R2Y = 0.941, Q2 = 0.652; Figure 3A). Permutation tests showed stability and robustness of the supervised models (Figure S4B). Differential metabolites were assigned based on VIP value > 1 and *p* < 0.05. Pathway enrichment analysis using Mummichog showed significant enrichment (*p* < 0.05) of several pathways related to caffeine metabolism, porphyrin metabolism, chondroitin sulfate degradation, heparan sulfate degradation, and vitamin H (biotin) metabolism, among others (Figure S4C), in BC samples compared with those in RCC samples. Overall, 24 differential metabolites were identified as shown in Table S7. ROC analysis showed that 9 metabolites have a potentially useful diagnostic value for BC and RCC discrimination (Table S8). Further metabolite panels were explored to achieve better predictive ability. Using Random Forest algorithms, a metabolite panel consisting of 7,8-Dihydropteroic acid, Avenoleic acid, and 3,4-Dimethyl-5-pentyl-2-furanundecanoic acid showed the best predictive ability with ROC area of 0.862 for the testing dataset (Figure S4D) and of 0.802 for the external validation dataset (Figure S4E) for BC and RCC discrimination.

#### Distinction BC and RCC by Plasma Lipidomics

Lipidomic profiling differentiation was explored between BC and RCC plasma samples using similar multiple statistic methods. PCA analysis also showed separation trend to some extent of BC and RCC (R2X = 0.602, Q2 = 0.272; Figure S5A). Further, the OPLS-DA model achieved better separation (R2X = 0.339, R2Y = 0.959, Q2 = 0.715; Figure 3B). Hundred permutation tests showed no over-fitting of the models (Figure S5B). Pathway enrichment analysis using Mummichog showed significant enrichment in pathways related to aspartate and asparagine metabolism, pentose phosphate pathway, hexose phosphorylation and vitamin H (biotin) metabolism, among others (Figure S5C), in BC samples compared with those in RCC samples. Further, a total of 17 differential metabolites were identified as shown in Table S9. Using Random Forest algorithms, a panel consisting of PS(P-38:0), 4E,14Z-Sphingadiene, Tetrapedic acid A (Table S10) showed the best predictive ability with ROC area of 0.853 for the testing dataset (Figure S5D) and 0.898 for the external validation dataset (Figure S5E) for BC and RCC discrimination.

#### Distinction BC and RCC by Combination of Plasma Metabolomics and Lipidomics

Combining the results of the identified differential metabolites, the relative intensity was plotted as a heatmap in Figure 3C. The up-regulated metabolites in BC compared to RCC included some acyl carnitines, fatty acids, amino acids, and derivatives and glycerophospholipids (GPs). The down-regulated metabolites included some dipeptides, sterol lipids (STs), sphingolipids (SPs), and fatty acyls (FAs) in BC compared with those in RCC. Multivariate ROC curve-based exploratory analysis was tried to achieve a better predictive model using these combined differential metabolites. A panel consisting of 7,8-Dihydropteroic acid, PS(P-38:0), 9,10,13-TriHOME, Avenoleic acid, 3,4-Dimethyl-5-pentyl-2-furanundecanoic acid and 4E,14Z-Sphingadiene (Table 3) showed the best predictive ability with ROC area of 0.939 for the testing dataset (Figure 3D) and 0.942 for the external validation dataset (Figure 3E).

**Table 3:** Differential metabolites for distinction of BC and RCC.

**Features**	**Metabolites ID**	**Description**	**Score**	***p*-value**	**Fold change (BC/RCC)**	**AUC**
1.15_297.1068m/z	HMDB01412	7,8-Dihydropteroic acid^**a**^	47.3	3.29E-04	3.41	0.8055
5.60_331.2470m/z	HMDB04710	9,10,13-TriHOME^**a**^	42.3	3.74E-05	4.93	0.7857
6.60_314.2448n	HMDB29978	Avenoleic acid^**a**^	39.5	1.47E-03	1.73	0.7556
4.85_372.2654n	HMDB31126	3,4-Dimethyl-5-pentyl-2-furanundecanoic acid^**a**^	53.1	2.61E-05	0.64	0.7300
8.39_826.5905m/z	LMGP03030046	PS(P-38:0)^**b**^	43.9	7.74E-07	0.45	0.7925
3.23_320.2539m/z	LMSP01080002	4E,14Z-Sphingadiene^**b**^	40.3	1.92E-04	0.6	0.7089
2.18_367.2823m/z	LMFA01050426	Tetrapedic acid A^**b**^	47.8	1.89E-04	0.34	0.7048

a *Metabolites identified by the chemical structure analysis matching with The Human Metabolome Database*.

b *Metabolites identified by the chemical structure analysis matching with LIPID MAPS*.

### Common Differential Metabolites for Differential Diagnosis Among BC, RCC, and Control

**Figure 4:**
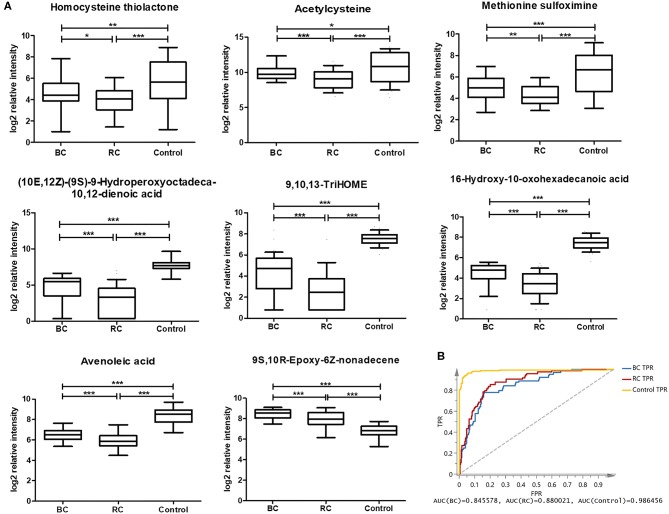
Analysis of 8 common differential metabolites in BC (64 samples), RCC (74 samples), and control (141 samples) group. Homocysteine thiolactone and acetylcysteine were confirmed by standard compounds. **(A)** Relative intensity of 8 common differential metabolites in BC, RCC, and control group. *, **, and *** represent *p*-value less than 0.05, 0.01, and 0.001 between two groups, respectively. **(B)** Score plot based on 8 common differential metabolites for BC, RCC, and control discrimination.

According to the above analysis, plasma metabolites could diagnose cancer (BC and RCC) from controls with high accuracy, and another panel of plasma metabolites could also discriminate BC and RCC with high accuracy. We further tried to find common differential metabolites among BC, RCC and control groups. Then, differential metabolites were selected in BC vs. control groups and RCC vs. control groups using similar multiple statistic methods as above. In all, 8 metabolites presented different levels in BC, RCC, and control groups. The relative content of the 8 metabolites in the BC, RCC, and control groups was plotted in Figure 4A. Non-parameter test was performed and the *p-*values from different groups were all <0.05, which showed in Figure 4A. Herein, homocysteine thiolactone, acetylcysteine, methionine sulfoximine, 9,10,13-TriHOME, avenoleic acid, (10E,12Z)-(9S)-9-Hydroperoxyoctadeca-10,12-dienoic acid, 16-Hydroxy-10-oxohexadecanoic acid were down-regulated in cancer groups compared with the control group, and the relative content in the RCC group was lower than that in the BC group. In addition, 9S,10R-Epoxy-6Z-nonadecene was up-regulated in the cancer groups compared with the control group, and the relative content in the RCC group was lower than that in the BC group. Further PCA score plot indicated that a panel of 8 common differential metabolites showed good predictive ability for BC, RCC and control discrimination, with an AUC of 0.8456 for the BC group, 0.88 for the RCC group, and 0.986 for the control group (Figure 4B).

## Discussion

Disease-related metabolomics is currently a hot area of research, and numerous metabolites have been proposed as potential biomarkers (5). Lipidomics, a specific component of metabolomics, has attracted increased attention due to its unique biological significance (38), and it is widely studied for the identification and validation of disease-specific biomarkers (12–14).

Within metabolomics, three analytical techniques are most used: nuclear magnetic resonance spectroscopy (NMR), and gas or liquid chromatography coupled to mass spectrometry (GC/MS and LC/MS, respectively), but they have different operational performance characteristics(Table S12). NMR is known for its reproducibility, minimal sample preparation requirements and its non-destructive nature, but MS methods possess much higher levels of sensitivity and are certainly more accessible to most laboratories (18, 39). While several metabolites cannot be analyzed by GC-MS because they are prone to thermal decomposition or are unable to be volatilized. In contrast, a LC-MS based platform can detect a wider range of chemical species, and reversed phase liquid chromatography (RPLC)-MS is the most widely used platform in metabonomic studies (27). Therefore, we applied RPLC-MS analysis in this study.

In this study, plasma metabolomics and lipidomics were utilized, first to explore potential biomarkers between cancer (BC and RCC) and non-cancer for early detection of genitourinary cancer (BC and RCC). Then, differential metabolites were explored between BC and RCC to find cancer-specific biomarker for differential diagnosis (Table 4). Furthermore, 8 common differential metabolites were also found that showed good predictive ability for BC, RCC, and control plasma sample discrimination.

**Table 4:** Performance of metabolomics/lipidomics panels for groups discrimination.

**ROC analysis**	**Plasma metabolomics**		**Plasma lipidomics**		**Combined plasma metabolomics and lipidomics**	
**Groups**	**Discovery group**	**Validation group**	**Discovery group**	**Validation group**	**Discovery group**	**Validation group**
Cancer vs. Control	0.985^**a**^	0.944^**a**^	0.993^**b**^	0.976^**b**^	1^**c**^	0.99^**c**^
BC vs. RCC	0.862^**d**^	0.802^**d**^	0.853^**e**^	0.898^**e**^	0.939^**f**^	0.942^**f**^

a *A panel consists of 9,10,13-TriHOME, 12,13-DHOME and linolenelaidic acid*.

b *A panel consists of 11Z-Eicosenal, 6Z-Heneicosen-9-one, behenic acid and 7Z-Tricosen-11-one*.

c *A panel consists of 9,10,13-TriHOME, 11Z-Eicosenal, 12,13-DHOME, 6Z-Heneicosen-9-one, linolenelaidic acid, behenic acid and 16-Hydroxy-10-oxohexadecanoic acid*.

d *A panel consists of 7,8-Dihydropteroic acid, Avenoleic acid and 3,4-Dimethyl-5-pentyl-2-furanundecanoic acid*.

e *A panel consists of PS(P-38:0), 4E,14Z-Sphingadiene and Tetrapedic acid A*.

f *A panel consists of 7,8-Dihydropteroic acid, PS(P-38:0), 9,10,13-TriHOME, Avenoleic acid, 3,4-Dimethyl-5-pentyl-2-furanundecanoic acid and 4E,14Z-Sphingadiene*.

BC and RC are two different types of genitourinary cancers differing in their cellular origins, which BC occurs on the mucous membrane of the bladder and RCC originates in the urinary tubular epithelial system of the renal parenchyma, thus, they represent distinct clinical entities (25, 40, 41). However, proteomics and metabolomics studies showed that similar pathway dysregulation could be found in both cancers, such as glycolysis, TCA cycle, fatty acid oxidation, etc (42, 43). We compared the main findings found in this study with previous reports (Table 5) and found some common dysregulation pathways, including glycolysis, lipid metabolism, and fatty acid beta-oxidation in BC and RCC patients. Among them, a massive shift in fatty acid metabolism and the carnitine shuttle was found in both cancers compared with that in the healthy controls. Fatty acids are involved in energy metabolism and cell membrane molecule synthesis (20). In tumors tissues, free fatty acids (FFA) are esterified to fatty acyl-CoAs and then transported into the mitochondria by carnitine palmitoyltransferase-1 (CPT1) and the carnitine system, while in normal tissue, they are subjected to b-oxidation as fatty acyl-CoAs to feed into the TCA cycle (42). Carnitine is essential in mediating the transport of acyl groups across the mitochondrial inner membrane (45). Disturbances in fatty acid metabolism and in the carnitine shuttle may contribute to energy metabolism disorders in cancer patients (42). Our metabolomics studies have led to the identification of carnitine derivatives as being significantly altered in the plasma of affected patients. This finding was validated *in vitro* using several RCC cell lines and show that these acylcarnitines, as a function of carbon chain length, affect cell survival, and markers of inflammation (46).

**Table 5:** The comparison of the main findings found in this study with previous related reports.

**Author (year)**	**Analytical platform**	**Sample type**	**BC^**a**^**	**RCC^**a**^**	**Control^**a**^**	**Pathways dysregulated in cancer compared to control**^**b**^				
						**Glycolysis**	**TCA cycle**	**Fatty acid beta-oxidation**	**Pentose phosphate pathway**	**Amino acid metabolism**	**Lipid metabolism**
Cao et al. (44)	NMR	Serum	37		45	↑				↓	↑
Jin et al. (23)	RPLC-MS	Urine	138		121	↑	↑	↑			
Wittmann et al. (19)	LC-MS and GC-MS	Urine	66		266	↑	↑			*	↑
Zhou et al. (20)	GC-MS	plasma	92		48		↑		↑	↑	↑
Kim et al. (16)	LC-MS and GC-MS	Urine		29	33	↑		↑		↓	
Lin et al. (17)	LC-MS	Serum		33	25			*		↓	*
Falegan et al. (18)	NMR and GC-MS	Urine and serum		40	13	↑	↑			*	*
Lin et al. (5)	LC-MS	Serum	24	24	24					*	*
Liu et al. (this study)	LC-MS	Serum	64	73	141			*	*	*	*

a *The number of patients recruited in the study*.

b Change trend of the Pathways dysregulated in cancer compared to control. (↑): up-regulated; (↓): down-regulated; (*): dysregulated.

In addition, linoleate metabolism was found to be disturbed in cancer samples compared with that in controls. Linoleate metabolism is involved in the generation of inflammatory mediators (47) and in the regulation of lipid metabolism by activation of the peroxisome proliferators-activated receptor alpha (PPARa) (48). 9,10,13-TriHOME is an important inflammatory mediator which has the ability to aggregate neutrophils (49). 12,13-DHOME is known to directly affect cell differentiation through its PPAR binding activity (50). Taken together, there is a common regulatory mechanism among these metabolic pathways that contributes to disturbances of energy supply, to inflammation, to activation of the immune response and to oxidative stress in cancer (BC and RCC) patients.

Though similar pathways dysregulations could be found in BC and RCC, significant different pathways also could be found between them, such as pentose phosphate pathway (22, 51), amino acid metabolism (43, 52). In this study, pathway analysis between BC and RCC showed disturbed aspartate and asparagine metabolism, pentose phosphate pathway, linoleic acid metabolism, and vitamin H (biotin) metabolism in BC compared with that in RCC. Pentose phosphate pathway (PPP) is a major pathway for glucose catabolism. Emerging evidence suggests that the PPP directly or indirectly provides reducing power to fuel the biosynthesis of lipids and nucleotides and sustains antioxidant responses to support cell survival and proliferation (53). Zhou et al. (20) also found that pentose phosphate pathway(PPP) were significantly upregulated in bladder cancer. Previous multi-omics analysis showed that pentose phosphate pathway, fatty acid b-oxidation, glutamine pathway and tryptophan metabolism are reprogrammed in RCC, and the changes are related to energy metabolism, oxidative stress and immunosuppression (42, 51, 54). These alterations in glucose metabolism and pentose phosphate pathway were in accordance with previous findings that oncogenic signaling pathways may promote cancer through rerouting the sugar metabolism (51, 53). (10E,12Z)-(9S)-9-Hydroperoxyoctadeca-10,12-dienoic acid and 9,10,13-TriHOME are involved in linoleic acid metabolism, and they are both up-regulated in BC compared with RCC. Linoleic acid has previously been reported to induce carcinogenesis through oxidative damage and pro-inflammatory mechanisms (55). Trihydroxyoctadecenoic acids (TriHOMEs) are linoleic acid-derived oxylipins with potential physiological relevance in inflammatory processes as well as in maintaining an intact skin barrier (56). 9,10,13-TriHOME is an important inflammatory mediator that has the ability to aggregate neutrophils (49), which suggested that inflammation may be higher in BC than in RCC. Previous mRNA expression analysis showed that BC samples showed strong immune expression signature, including T cell markers and inflammation genes (57). Inflammation occurs during all stages of the tumor and inflammation establishes cancer invasion metastasis by reducing apoptosis and increasing angiogenesis (58, 59).

In this study, 8 metabolites were found to show different levels in BC, RCC, and control groups. The relative intensity results (Figure 4A) showed that the 8 metabolites were significantly statistical different between the two kinds of cancers and control group, though the difference between the BC and RCC was less obvious. Herein, homocysteine thiolactone, acetylcysteine, and methionine sulfoximine are amino acids. 16-Hydroxy-10-oxohexadecanoic acid and 9S,10R-Epoxy-6Z-nonadecene are fatty acids that are involved in lipid transport and fatty acid metabolism. (10E,12Z)-(9S)-9-Hydroperoxyoctadeca-10,12-dienoic acid, avenoleic acid and 9,10,13-TriHOME are linoleic acids and their derivatives. Homocysteine (Hcy) was converted to Hcy-thiolactone by methionyl-tRNA synthetase (60). The relevance of cysteine metabolism in cancer has been reported, but these reports have been largely focused on its role in generating the antioxidant glutathione (61). Linoleic acid metabolites have previously been reported to have relevance in inflammatory processes (49, 55, 56), and 9S,10R-Epoxy-6Z-nonadecene is one of the unsaturated fatty acid metabolites. The 9S,10R-Epoxy-6Z-nonadecene level in the cancer group was obviously higher than that in the control group, and the relative content in the BC group was higher than that in the RCC group, as shown in Figure 4A. That finding was consistent with previous results that saturated fatty acyls decrease and that highly unsaturated fatty acyls increase in tumor tissues (30). However, the specific biological function of 9S,10R-Epoxy-6Z-nonadecene remains to be uncovered.

Among the above 8 metabolites, though the fold changes of two metabolites (Avenoleic acid and 9S,10R-Epoxy-6Z-nonadecene) in BC and RCC distinguish were <1.5, their performances in the difference between BC or RCC and control were better (Table S11). Moreover, the PCA score plot of the panel consisting of these 8 metabolites showed good predictive ability for BC, RCC, and control discrimination, with an AUC of 0.8456 for the BC group, 0.88 for the RCC group and 0.986 for the control group. Therefore, the panel of 8 common differential metabolites might be used as potential biomarker for early detection of BC and RCC from control. On the other hand, present study was a relative small sample size and single-center pilot study, further larger sample cohorts and multiple-center study will be performed in the future for more comprehensive validation. The prediction of prognosis after surgery was an important issue for clinical research. The performances of the panel on this issue need to be evaluated by follow-up data in the future.

## Limitation of this Study

The results of BC and RCC plasma metabolome in this study indicated that it was feasible to utilize plasma metabolomics and lipidomics for discriminating cancer from non-cancer and for differential diagnose of BC and RCC. However, this study also has the following limitations to be considered. (1) The sample size of the present study was relatively small, further larger sample cohorts and multiple-center study should be performed for more comprehensive validation. (2) In this study, the differential metabolites were discovered by non-targeted LC/MS/MS analysis. This approach provided a preliminary result in potential candidate biomarkers. To validate the above results, a targeted approach with authentic standards should be used in future validation study. (3) The samples recruited in this study were only from non-metastatic stage, thus the grades and stages of cancer were not taken into consideration. Whether different grades and stages of cancer will present different serum metabolomic pattern or not is of great importance, which should be thoroughly evaluated by a large-scale cohort in the future. (4) Due to the short follow-up time of the cohort in this study, we could not evaluate the relationship of the differential metabolites and clinical parameters, which should be comprehensively analyzed in future work. (5) In this study, the potential metabolite biomarkers of BC and RCC were discovered, but their function and mechanism in cancers had not been investigated, which should be presented by cell lines or animal model analysis in the future.

## Conclusion

In conclusion, we have for the first time utilized data from a combination of plasma metabolomics and lipidomics analysis for BC and RCC early detection and screening, and provided a new insight into the differential diagnosis of BC and RCC. The results suggested that the plasma metabolome and lipidome could differentiate BC and RCC patients from controls, and panels of plasma metabolites were discovered to have potential value for BC and RCC discrimination. Moreover, the results suggested that combining plasma metabolomics and lipidomics has better predictive performance than either method alone. We also identified 8 metabolites might be used as potential biomarker panel to distinguish BC, RCC, and control.

## Data Availability Statement

All datasets generated for this study are included in the article/Supplementary Material.

## Ethics Statement

The studies involving human participants were reviewed and approved by Institutional Review Board of the Institute of Basic Medical Sciences, Chinese Academy of Medical Sciences. The patients/participants provided their written informed consent to participate in this study. Written informed consent was obtained from the individual(s) for the publication of any potentially identifiable images or data included in this article.

## Author Contributions

XianL, MZ, and XC prepared the first draft. XiaoL and WS conceived and designed the experiments. JL, XT, and ZW performed the experiments. XianL, XiaoL, HS, and ZG analyzed the data. MZ, XC, YZ, and ZJ contributed to collect clinical samples. All authors approved the final manuscript.

## Conflict of Interest

The authors declare that the research was conducted in the absence of any commercial or financial relationships that could be construed as a potential conflict of interest.
