# Supplementary material for: LC-MS-Based Plasma Metabolomics and Lipidomics Analyses for Differential Diagnosis of Bladder Cancer and Renal Cell Carcinoma
Source: Front Oncol. 2020 May 15;10:717. doi: 10.3389/fonc.2020.00717 (PMC7243740; doi:10.3389/fonc.2020.00717)

Supplementary Information

LC-MS based plasma metabolomics and lipidomics analyses for differential diagnosis of bladder cancer and renal cell carcinoma

Xiang Liu[1#], Mingxin Zhang[2,3#], Xiangming Cheng[2#], Xiaoyan Liu[1], Haidan Sun[1], Zhengguang Guo[1], Jing Li[1], Xiaoyue Tang[1], Zhan Wang[2], Wei Sun[1*], Yushi Zhang[2*], Zhigang Ji[2*]

[1] Institute of Basic Medical Sciences, Chinese Academy of Medical Sciences, School of Basic Medicine, Peking Union Medical College, Beijing, China

[2] Department of Urology, Peking Union Medical College Hospital, Chinese Academy of Medical Science, Beijing, China

[3]Department of Urology, The Affiliated Hospital of Qingdao University,Qingdao, China

***Corresponding author:** Prof. Wei Sun, E-mail: sunwei1018@sina.com; Tel: 0086-010-69156995
Prof. Yushi Zhang, E-mail: zhangyushi2014@126.com; Tel.: 0086-010-69152529
Zhigang Ji, MD, E-mail: cxm19900925@126.com; Tel: 0086-010-69152529

[#] These authors contributed equally to this work

Supplementary Methods

1. Sample Preparation
2. LC-MS Analysis
3. Data processing using Progenesis QI
4. Confirmation of compounds characterization
5. Statistical data analysis
6. Metabolite annotation and pathway analysis

Supplementary Tables

Table S1 The detailed clinical information for each patients who participated in this study.

Table S2 Statistical metrics of individually analyzed on PCA and OPLS-DA model

Table S3 Differential metabolites between cancer (BC and RCC) and health control on plasma metabolomics.

Table S4 AUC value of differential metabolites for cancer (BC and RCC) distinction on plasma metabolomics.

Table S5 Differential lipids between cancer (BC and RCC) and health control on plasma lipidomics.

Table S6 AUC value of differential lipids for cancer (BC and RCC) distinction on plasma lipidomics.

Table S7 Differential metabolites between BC and RCC on plasma metabolomics.

Table S8 AUC value of differential metabolites for BC and RCC distinction on plasma metabolomics.

Table S9 Differential lipids between BC and RCC on plasma lipidomics.

Table S10 AUC value of differential lipids for BC and RCC distinction on plasma lipidomics.

Table S11 Eight common differential metabolites in BC (64 samples), RCC (74 samples) and control (141 samples) group.

Table S12 The advantages and limitations of NMR spectroscopy and MS spectrometry as an analytical tool for metabolomics research.

Supplementary Figures

Fig. S1 Assessment of QC samples. **a.** Trend plot showing the variation of t [1] over all QC Samples on plasma metabolomics. X axis numbers represented sample number, Y axis was arbitrary (3 s.d.); **b.** Trend plot showing the variation of t [1] over all QC Samples on plasma lipidomics. **c.** PC1 versus PC2 of test samples and QC on plasma metabolomics. **d.** PC1 versus PC2 of test samples and QC on plasma lipidomics.

Fig. S2 Analysis of metabolic profiling variation of 95 cancer (BC and RCC) samples and 95 control samples. **a.** Score plot of unsupervised PCA overview of plasma metabolic profiling between cancer and control. **b.** 100 permutation tests of the OPLS-DA model based on cancer and control plasma metabolomics. **c.** Top five shifted metabolic pathways in cancer (BC and RCC) compared with control. **d.** ROC plot with discovery group for distinction of cancer and control based on 9,10,13-TriHOME, 12,13-DHOME and linolenelaidic acid. **e.** ROC plot with external validation group for distinction of 43 cancer samples and 46 control samples based on 9,10,13-TriHOME, 12,13-DHOME and linolenelaidic acid.

Fig. S3 Analysis of lipidomic profiling variation of 95 cancer (BC and RCC) samples and 95 control samples. **a.** Score plot of unsupervised PCA overview of plasma lipidomic profiling between cancer and control. **b.** 100 permutation tests of the OPLS-DA model based on cancer and control plasma lipidomics. **c.** Top five shifted lipidomic pathways in cancer (BC and RCC) compared with control. **d.** ROC plot with discovery group for distinction of cancer and control based on 11Z-Eicosenal, 6Z-Heneicosen-9-one, behenic acid and 7Z-Tricosen-11-one. **e.** ROC plot with external validation group for distinction of 43 cancer samples and 46 control samples based on 11Z-Eicosenal, 6Z-Heneicosen-9-one, behenic acid and 7Z-Tricosen-11-one.

Fig. S4 Analysis of metabolic profiling variation of 42 BC samples and 53 RCC samples. **a.** Score plot of unsupervised PCA overview of plasma metabolic profiling between BC and RCC. **b.** 100 permutation tests of the OPLS-DA model based on BC and RCC plasma metabolomics. **c.** Top five shifted metabolic pathways in BC and RCC. **d.** ROC plot with discovery group for distinction of BC and RCC based on 7,8-Dihydropteroic acid, Avenoleic acid and 3,4-Dimethyl-5-pentyl-2-furanundecanoic acid. **e.** ROC plot with external validation group for distinction of 22 BC samples and 21 RCC samples based on 7,8-Dihydropteroic acid, Avenoleic acid and 3,4-Dimethyl-5-pentyl-2-furanundecanoic acid.

Fig. S5 Analysis of lipidomic profiling variation of 42 BC samples and 53 RCC samples. **a.** Score plot of unsupervised PCA overview of plasma lipidomic profiling between BC and RCC. **b.** 100 permutation tests of the OPLS-DA model based on BC and RCC plasma lipidomics. **c.** Top five shifted lipidomic pathways in BC and RCC. **d.** ROC plot with discovery group for distinction of BC and RCC based on PS(P-38:0), 4E,14Z-Sphingadiene and Tetrapedic acid A. **e.** ROC plot with external validation group for distinction of 22 BC samples and 21 RCC samples based on PS(P-38:0), 4E,14Z-Sphingadiene and Tetrapedic acid A.

Fig. S6 Typical base peak chromatograms from representative serum samples (a) Typical base peak chromatograms of plasma metabolomics. Top：control sample; Middle: Renal cell carcinoma sample; Down: bladder cancer sample. (b) Typical base peak chromatograms of plasma lipidomics. Top：bladder cancer sample; Middle: control sample; Down: Renal cell carcinoma sample.

Fig. S7 MS2 spectra of 5 metabolites identified in standard compounds (STD) and in QC sample. (a) hypoxanthine, (b) homocysteine thiolactone, (c) 4-Ethylphenol, (d) L-octanoylcarnitine, (e) acetylcysteine

Supplementary Methods

Sample collection and preparation

The consent procedure and the research protocol for this study were approved by the Institutional Review Board of the Institute of Basic Medical Sciences, Chinese Academy of Medical Sciences (Project NO: 047-2019). And all human subjects provided informed consent and took a series of physical examinations and laboratory tests before participating in this study, including blood pressure, body mass index (BMI), fasting blood glucose (FBG), total cholesterol (TC), triglyceride (TG) etc. Finally, a total of 141 participants aged 27 to 74 years with health standard were recruited in this study. Meanwhile, the BC and RCC patients also took above tests, and only the patients with normal results were recruited.

The plasma samples from 64 bladder cancer (BC) patients, 74 renal cell carcinoma (RCC) patients and 141 healthy controls were collected from Peking Union Hospital (Table 1, the detailed clinical information was shown in Table S1). All the plasma samples in our study were collected before any treatments. The plasma samples were collected in the morning from 07:00 a.m.–09:00 a.m. after an overnight fast to eliminate dietary disturbances. After collected, all plasma samples were separated following centrifugation at 1,024 g for 10 min at 4ºC and were stored at –80ºC.

Sample Preparation

For plasma metabolomics, fifty microliters of sample were mixed with 150 μL of H_2O by vortexed for 30 s to dilute the sample, then acetonitrile (400 μL) was added into each sample (200 μL), the mixture was vortexed for 1 min, stand for 30 min at -20 ºC and centrifuged at 14,000 × g for 10 min. The supernatant was dried under vacuum and then reconstituted with 100 μL of 2% acetonitrile. Plasma metabolites were further separated from larger molecules using 10 kDa molecular weight cut-off ultracentrifugation filters (Millipore Amicon Ultra, MA) before transferred to the autosamplers. The quality control (QC) [1] sample was a pooled sample prepared by mixing aliquots of two hundred randomly selected samples across different groups to be analyzed and therefore globally representative of the whole sample set. The QC samples were injected every ten samples throughout the analytical run to provide a set of data from which method stability and repeatability can be assessed.

For plasma lipidomics, 200 μL plasma samples were precipitated by addition of 600 μL of isopropanol (IPA) precooled to −20 °C. Samples were vortex mixed for 1 min. After 10 min of incubation at room temperature, samples were stored 2h at −20 °C to improve protein precipitation and then centrifuged at 14, 000g for 20 min. The supernatant was dried under vacuum and then reconstituted with 100 μL of 50% IPA. QC sample was prepared the same as plasma metabolomics QC sample.

LC-MS Analysis

Ultra-performance LC-MS analyses of samples were conducted using a Waters ACQUITY H-class LC system coupled with an LTQ-Orbitrap Velos mass spectrometer

(Thermo Fisher Scientific, MA. USA). Plasma metabolites were separated with an 18 min gradient on a Waters HSS C18 column (3.0× 100 mm, 1.7μm) at a flow rate of 0.5 mL/min. Mobile phase A was 0.1% formic acid in H2O and mobile phase B was acetonitrile. The gradient was set as follows: 0–1 min, 2% solvent B; 1–3min, 2–55% solvent B; 3–8 min, 55-100% solvent B; 8-13 min, 100% solvent B; 13-13.1 min, 100-2% solvent B; 13.1-18 min, 2% solvent B. The column temperature was set as 50°C. Plasma lipids were separated with a 23 min gradient on a Waters HSS C18 column (3.0× 100 mm, 1.7μm) at a flow rate of 0.4 mL/min. Mobile phase A was 10 mM ammonium acetate in acetonitrile (4:6) and mobile phase B was 10 mM ammonium acetate in isopropanol/acetonitrile (9:1). The gradient was set as follows: 0 min, 40% solvent B; 0-2 min, 40-43% solvent B; 2-8 min, 43-85% solvent B; 8-15 min, 85-99% solvent B; 15-18 min, 99% solvent B; 18-18.1 min, 99-40% solvent B; 18.1-23 min, 40% solvent B. The column temperature was set as 55°C.

The mass spectrometer was operated in positive ion mode using the m/z range 100–1000 m/z at a resolution of 60 K. Automatic gain control (AGC) target was 1×10^6 and maximum injection time (IT) was 100 ms. Subsequently differential metabolites identification was performed by UPLC targeted-MS/MS analyses of QC sample. It acquired at a resolution of 15 K with AGC target of 5×10^5, maximum IT of 50 ms, and isolation window of 3 m/z. Collision energy was optimized as 20, 40, 60 for each target with higher-energy collisional dissociation (HCD) fragmentation.

Data processing using Progenesis QI

Raw data files were processed by the Progenesis QI 2.2 (Waters, Milford, MA, USA) software [2]. The detailed workflow for data processing facilitated by Progenesis QI is involved “create a new experiment”, “import data”, “review alignment”, “experiment design setup”, “peak picking”, “reviewed convolution”, and “identify compounds” in sequence. In general, the whole process ran automatically using optimized parameter settings. (1) In the stage of create a new experiment, adduct ion was carefully selected as it would influence the number of characterized compounds and also the identification accuracy. Based on the ionization behaviors of reference standards, the adduct ion forms, comprising [M + H]+, [M + Na]+, [M + K], [M + NH4]+, [2M + H]+, [2M + Na]+,[2M + NH4]+, [M + H – H2O]+ and [M + H – 2H2O]+ , were selected. (2) The MS data acquired by LC-MS for all the plasma samples were imported into the Progenesis QI software, generating a 2D ion intensity map with the retention time and m/z information as the ordinate and abscissa, respectively. (3) Peak alignment was carried out in automatic manner taking a QC run as the reference, the score values for all the samples were greater than 90 %. (4) For peak picking, the thresholds of chromatographic peak absolute intensity, and retention time limits can be set to achieve the maximum real ion signals with noise excluded. In the present study, absolute intensity and retention time limit were set at 1000 and default. (5) Further compound identification was performed by searching the HMDB database 4.0 [3]. The identification results combined with the intensity data were exported as .csv files for subsequent compound confirmation and multivariate statistical analysis.

Confirmation of compounds characterization

Detailed compound identification information (.csv file) included compound ID, adducts, formula, score, fragmentation score, mass error (in ppm), isotope similarity, theoretical isotope distribution, web link, and m/z values. The data was further analyzed in detail, under which more abundant MS/MS fragments were acquired. Confirmation of the differential compounds was performed by the parameters, including Score, Fragmentation score, and Isotope similarity given by Progenesis QI. Score ranging from 0 to 60, is used to quantify the reliability of each identity. According to the score results of the reference standards, the threshold was set at 35.0. Fragmentation score represents the matching degree between the theoretical fragments and the measured ones. The fragmentation score of 0 indicates no match occurs or the compound generates no fragments. Isotope similarity is calculated by comparison of the measured isotope distribution of a precursor ion with the theoretical. The compound identification is more reliable the higher the values obtained. In addition, Homocysteine thiolactone, Hypoxanthine, 4-Ethylphenol, L-Octanoylcarnitine and Acetylcysteine were confirmed by standard compounds.

Statistical data analysis

Further data pre-processing including missing value estimation, Log transformation and Pareto scaling were carried out to make features more comparable using MetaboAnalyst 4.0 [4] (http://www.metaboanalyst.ca). Features existed in at least 50% samples, and the features with coefficient of variation (CV) less than 30% in the QC samples were further analyzed. Then, non-parametric tests (Wilcoxon rank-sum test) was used to evaluate the significance of variables. And adjusted P-value (FDR) cutoff was set as 0.05. Pattern recognition analysis (principal component analysis, PCA; orthogonal partial least squares discriminant analysis, OPLS-DA) was carried out using SIMCA 14.0 (Umetrics, Sweden) software. The selected differential variables must meet the following three conditions: 1) adjusted P-value <0.05; 2) Fold change between two groups >1.5; 3) VIP value obtained from OPLS-DA was above 1. Identified differential metabolites were subjected to MetaboAnalyst 4.0 to perform exploratory ROC analysis. Random Forest algorithms were used for ROC curve construction. Box plots showed values of median, minimum, maximum and the variability of data sets using the first and third quartiles. Statistically significant differences in graphs were calculated using the Mann-Whitney test in GraphPad Prism 6 software (GraphPad Software, Inc., La Jolla, CA, USA), where *, **, and *** represent p-values less than 0.05, 0.01, and 0.001 between two groups, respectively.

Metabolite annotation and pathway analysis

Metabolic pathways and predicted metabolites in the pathways were analyzed using Mummichog [5]. Mummichog is a program written in python for analyzing data from high-throughput, untargeted HRLC-MS metabolomics, bypassing the tedious and challenging metabolite identification. It leverages the organization of metabolic networks to predict functional pathways directly from feature tables and generate a list of tentative metabolites annotations through functional activity analysis. We input tab-delimited text files of peaks list with m/z, retain time, P value, and log2(FC) of two group analysis into Mummichog to conduct the pathways

and module analysis. KEGG human network model was selected, and the cut-off P value was set to 0.05 to generate a list of significant features. The analytical mode of mass spec was set to positive according the data source. Other options remained the default. Results from annotation, pathway analysis, and network module analysis were given. We then used MetaboAnalyst 4.0 [4] (http://www.metaboanalyst.ca) to visualize the results files of the metabolic pathways network.

References :

1 Sangster, T., Major, H., Plumb, R., Wilson, A. J. & Wilson, I. D. A pragmatic and readily implemented quality control strategy for HPLC-MS and GC-MS-based metabonomic analysis. *The Analyst* **131**, 1075-1078, doi:10.1039/b604498k (2006).

2 Zhang, J. *et al.* An intelligentized strategy for endogenous small molecules characterization and quality evaluation of earthworm from two geographic origins by ultra-high performance HILIC/QTOF MS(E) and Progenesis QI. *Analytical and bioanalytical chemistry* **408**, 3881-3890, doi:10.1007/s00216-016-9482-3 (2016).

3 Wishart, D. S. *et al.* HMDB 4.0: the human metabolome database for 2018. *Nucleic acids research* **46**, D608-d617, doi:10.1093/nar/gkx1089 (2018).

4 Chong, J. *et al.* MetaboAnalyst 4.0: towards more transparent and integrative metabolomics analysis. *Nucleic acids research* **46**, W486-w494, doi:10.1093/nar/gky310 (2018).

5 Li, S. *et al.* Predicting network activity from high throughput metabolomics. *PLoS Comput Biol* **9**, e1003123, doi:10.1371/journal.pcbi.1003123 (2013).

Supplementary Tables

Table S1. The detailed clinical information for each patient who participated in this study

The detailed clinical information for each patient with bladder cancer									
Discovery group					Validation group				
No.	Sex	Age	Pathology	Pathological grade	No.	Sex	Age	Pathology	Pathological grade
B101	male	57	NMIBC[1]	Low	BC76	male	63	NMIBC	Low
B103	male	33	NMIBC	Low	BC113	male	66	NMIBC	High
B136	male	82	NMIBC	Low	BC114	male	54	NMIBC	High
B139	male	67	NMIBC	High	BC13	male	65	NMIBC	Low
B144	male	68	NMIBC	High	BC142	male	86	NMIBC	Low
BC101	male	75	NMIBC	High	BC143	male	44	NMIBC	High
BC106	male	47	NMIBC	Low	BC19	male	60	NMIBC	Low
BC114	male	54	NMIBC	High	BC24	male	75	NMIBC	Low
BC115	male	60	NMIBC	High	BC31	male	76	NMIBC	High
BC132	male	55	NMIBC	Low	BC32	male	64	NMIBC	High
BC124	male	62	NMIBC	High	BC50	male	31	NMIBC	Low
BC125	male	62	NMIBC	High	BC57	male	59	NMIBC	Low
BC129	male	53	NMIBC	High	BC89	male	82	NMIBC	High
BC135	male	59	NMIBC	High	BC93	male	68	NMIBC	Low
BC140	male	73	NMIBC	Low	BC121	female	58	NMIBC	Low
BC144	male	71	NMIBC	Low	BC21	female	65	NMIBC	High
BC17	male	81	NMIBC	High	BC29	female	52	NMIBC	Low
BC18	male	69	NMIBC	Low	BC30	female	56	NMIBC	High
BC37	male	62	NMIBC	High	BC48	female	69	NMIBC	Low
BC41	male	28	NMIBC	High	BC5	female	71	NMIBC	Low
BC45	male	60	NMIBC	High	BC77	female	69	NMIBC	High
BC46	male	67	NMIBC	High	BC87	female	63	NMIBC	High
BC49	male	69	NMIBC	High					

BC53	male	75	NMIBC	High
BC58	male	41	NMIBC	High
BC59	male	59	NMIBC	Low
BC64	male	69	NMIBC	High
BC82	male	85	NMIBC	Low
BC83	male	58	NMIBC	High
BC84	male	63	NMIBC	Low
BC116	male	65	NMIBC	Low
BC107	female	45	NMIBC	High
BC108	female	48	NMIBC	Low
BC109	female	65	NMIBC	High
BC126	female	62	NMIBC	Low
BC133	female	66	NMIBC	High
BC145	female	62	NMIBC	High
BC39	female	80	NMIBC	Low
BC54	female	76	NMIBC	High
BC61	female	87	NMIBC	Low
BC72	female	72	NMIBC	High
BC96	female	38	NMIBC	High

The detailed clinical information for each patient with renal cell carcinoma

**Discovery group**					**Validation group**				
**No.**	**Sex**	**Age**	**Pathology**	**Pathological stage**	**No.**	**Sex**	**Age**	**Pathology**	**Pathological stage**
R939	male	77	clear cell	T1a[2]	R1119	male	55	clear cell	T1a
R941	female	49	clear cell	T1a	R1120	male	53	clear cell	T1b
R942	male	60	clear cell	T1a	R1128	male	56	clear cell	T1a
R943	male	45	clear cell	T1a	R1130	male	45	clear cell	T3a
R944	male	58	clear cell	T1b[3]	R1132	male	43	clear cell	T1a
R945	male	78	clear cell	T3a	R1136	male	65	clear cell	T1b
R946	male	54	clear cell	T1a	R1139	male	24	clear cell	T1a
R950	male	53	clear cell	T1a	R1142	male	48	clear cell	T1a

R951	male	47	clear cell	T3a
R952	male	73	clear cell	T3a
R954	male	42	clear cell	T1a
R955	female	65	clear cell	T1a
R959	male	63	clear cell	T1a
R964	female	71	papillary	T1a
R965	female	72	clear cell	T1a
R966	female	57	clear cell	T1a
R967	male	44	clear cell	T1a
R970	male	82	clear cell	T1a
R972	male	48	papillary	T1a
R973	male	45	clear cell	T1a
R975	male	73	clear cell	T3a
R976	male	69	clear cell	T1a
R977	female	67	clear cell	T1b
R982	female	74	clear cell	T3a
R985	male	84	clear cell	T1b
R986	male	56	clear cell	T1b
R989	female	57	papillary	T1a
R990	male	59	clear cell	T2a[4]
R993	female	55	clear cell	T3a
R994	female	15	papillary	T3a[5]
R995	female	68	clear cell	T1a
R996	male	47	clear cell	T3a
R997	male	65	clear cell	T3a
R998	male	57	papillary	T1a
R1006	female	67	clear cell	T3a
R1008	female	64	clear cell	T3a
R1010	male	40	papillary	T3a
R1027	male	80	clear cell	T1b
R1162	male	60	clear cell	T1b
R1164	male	35	clear cell	T1a
R1165	male	52	clear cell	T1a
R1169	male	56	clear cell	T1b
R1171	male	66	clear cell	T1b
R1173	male	45	clear cell	T1a
R1182	male	65	Papillary	T1a
R1186	male	64	clear cell	T1b
R1137	female	50	chromophobe	T1b
R1140	female	63	clear cell	T2a
R1144	female	24	clear cell	T1a
R1152	female	41	clear cell	T1b
R1172	female	54	clear cell	T2a

R1042	male	48	clear cell	T1a
R1043	female	50	clear cell	T2a
R1051	male	50	clear cell	T1b
R1070	male	65	clear cell	T1b
R1074	female	33	chromophobe	T1b
R1077	male	66	clear cell	T1b
R1078	male	52	clear cell	T1b
R1079	male	59	clear cell	T1b
R1086	male	44	papillary	T3a
R1088	male	53	clear cell	T1a
R1090	female	50	clear cell	T3a
R1096	male	68	clear cell	T1b
R1098	male	14	clear cell	T1b
R1100	female	30	clear cell	T1b
R1134	male	50	clear cell	T1b

[1]NMIBC: nonmuscle- invasive bladder cancer; [2]T1a: tumour ≤4 cm and confined to kidney; [3]T1b: tumour >4 cm and ≤7 cm, and confined to kidney; [4]T2: tumour >7 cm and confined to kidney; [5]T3a: tumour invades adrenal gland or perinephric fat but not beyond Gerota's fascia;

Table S2 Statistical metrics of individually analyzed on PCA and OPLS-DA model.

Model Type	R2X(cum)	R2Y(cum)	Q2(cum)	CV-ANOVA *p*-Value	CV-ANOVA F
**Distinction cancer (BC and RCC) from control by plasma metabolomics**					
PCA	0.624		0.416		
OPLS-DA	0.263	0.953	0.931	0	408.19
**Distinction cancer (BC and RCC) from control by plasma lipidomics**					
PCA	0.682		0.406		
OPLS-DA	0.296	0.949	0.924	0	372.939
**Distinction BC and RCC by plasma metabolomics**					
PCA	0.557		0.324		
OPLS-DA	0.322	0.941	0.652	8.14e-017	20.1704
**Distinction BC and RCC by plasma lipidomics**					
PCA	0.602		0.272		
OPLS-DA	0.339	0.959	0.715	5.68e-019	21.0664

Table S3. Differential metabolites between cancer (BC and RCC) and health control on plasma metabolomics

Features	Metabolites ID	Description	Score	*p*-value	Fold Change （cancer/HC）
1.56_191.1515n	HMDB31513	3-Hydroxynonanoic acid	38.1	6.85E-16	20.31
4.52_260.0209m/z	HMDB02028	DOPA sulfate	39	2.69E-27	12.78
4.52_107.0850m/z	HMDB31414	2,3-Dimethyl-2-cyclohexen-1-one	39.2	2.89E-27	11.53
1.04_495.1913m/z	HMDB28829	Glutamyl-Threonine	46	1.07E-22	8.47
1.11_226.1044m/z	HMDB62176	N-lactoyl-Leucine	42	1.43E-20	4.82
5.84_488.2677m/z	HMDB32799	(R)-1-O-[b-D-Glucopyranosyl-(1->6)-b-D-glucopyranoside]-1,3-octanediol	48.8	1.61E-09	3.84
4.52_105.0694m/z	HMDB29306	4-Ethylphenol	39	1.68E-24	3.34
5.84_414.2991m/z	HMDB62332	N-Docosahexaenoyl GABA	41.3	5.21E-06	2.62
5.84_449.3128n	HMDB00631	Deoxycholic acid glycine conjugate	56.2	1.48E-05	2.47
4.73_288.2161m/z	HMDB00791	L-Octanoylcarnitine	51.7	9.30E-14	0.63
6.72_251.1633m/z	HMDB37212	Menthyl lactate	41.2	1.66E-15	0.58
5.15_316.2474m/z	HMDB62631	O-decanoyl-L-carnitine	49.5	2.81E-14	0.57
1.89_136.0380n	HMDB00157	Hypoxanthine	41.1	8.60E-14	0.54
5.15_257.1739m/z	HMDB00394	3-Hydroxytetradecanedioic acid	39.6	2.34E-13	0.52
5.09_360.2733m/z	HMDB13164	2-Hydroxylauroylcarnitine	40.6	2.88E-13	0.52
4.37_304.2107m/z	HMDB61634	3-hydroxyoctanoyl carnitine	46.4	1.84E-15	0.46
6.59_279.2309m/z	HMDB30964	Linolenelaidic acid	38.1	5.15E-23	0.42
0.99_219.0965m/z	HMDB06248	5-L-Glutamyl-L-alanine	50.2	1.60E-21	0.31
7.16_312.2292n	HMDB62434	9-Hydroperoxyoctadeca-10,12-dienoic acid	44.6	6.20E-26	0.28
4.48_184.0730n	HMDB36072	Genipic acid	42.7	1.34E-13	0.19
6.60_314.2448n	HMDB04705	12,13-DHOME	42.7	6.90E-27	0.18
5.30_330.2628m/z	HMDB13321	Undecanoylcarnitine	51.5	2.12E-17	0.18
5.28_267.1218m/z	HMDB32472	Polyethylene, oxidized	46.5	1.32E-17	0.14
5.60_331.2470m/z	HMDB04710	9,10,13-TriHOME	42.3	1.09E-28	0.10
5.81_269.2104m/z	HMDB41287	16-Hydroxy-10-oxohexadecanoic acid	45.3	2.97E-29	0.1

Table S4. AUC value of differential metabolites for cancer (BC and RCC) distinction on plasma metabolomics

Name	AUC
16-Hydroxy-10-oxohexadecanoic acid	0.995
9,10,13-TriHOME	0.985
2,3-Dimethyl-2-cyclohexen-1-one	0.972
DOPA sulfate	0.970
12,13-DHOME	0.968
9-Hydroperoxyoctadeca-10,12-dienoic acid	0.959
4-Ethylphenol	0.946
Linolenelaidic acid	0.931
Glutamyl-Threonine	0.925
5-L-Glutamyl-L-alanine	0.915
N-lactoyl-Leucine	0.905
Polyethylene, oxidized	0.872
Undecanoylcarnitine	0.869
3-Hydroxynonanoic acid	0.852
Menthyl lactate	0.847
3-hydroxyoctanoyl carnitine	0.847
O-decanoyl-L-carnitine	0.832
Hypoxanthine	0.826
L-Octanoylcarnitine	0.825
Genipic acid	0.823
3-Hydroxytetradecanedioic acid	0.820
2-Hydroxylauroylcarnitine	0.819
R-1-O-b-D-Glucopyranosyl-1-6-b-D-glucopyranoside-1,3-octanediol	0.765
N-Docosahexaenoyl GABA	0.703

Table S5. Differential lipids between cancer (BC and RCC) and health control on plasma lipidomics

Features	Metabolites ID	Description	Score	*p*-value	Fold Change（cancer/HC）
9.09_311.3170n	LMFA06000248	11Z-Eicosenal	50.9	5.89E-27	2.26
9.24_325.3325n	LMFA12000215	6Z-Heneicosen-9-one	41.5	1.27E-24	2.25
8.12_298.3087m/z	LMFA12000203	10Z-Nonadecen-2-one	47.6	6.07E-11	1.66
9.71_354.3710m/z	LMFA12000222	7Z-Tricosen-11-one	48.7	7.95E-12	1.65
10.40_718.5334m/z	LMGP02010010	PE (34:1)	44	7.08E-04	1.61
4.49_502.3708m/z	LMST01010327	(25S)-5alpha-cholestan-3beta,6alpha,7beta,8beta,15alpha,16beta,26-heptol	39.5	7.92E-06	1.58
11.06_339.2872m/z	LMFA01170036	Japanic acid	42.4	1.38E-07	1.52
5.88_374.3005m/z	LMFA08020028	N,N-(2,2-dihydroxy-ethyl) arachidonoyl amine	50.9	9.51E-05	0.66
4.45_432.3578n	LMST01031125	22,23-dihydroxycampesterol	47.6	3.92E-06	0.63
5.52_561.4091m/z	LMFA03050002	12S-HHTrE	47	4.30E-04	0.63
6.63_473.3573m/z	LMST03020675	1alpha,25-dihydroxy-2alpha-(3-hydroxypropoxy) vitamin D3	51.1	3.55E-04	0.59
7.46_405.3703m/z	LMFA01020350	24:0(17Me)	48.7	1.72E-06	0.57
5.52_491.3676m/z	LMST01031065	6beta-acetoxy-24-methylcholestan-3beta,5alpha,22R,24-tetrol	38.9	2.70E-06	0.56
6.47_405.3704m/z	LMFA01020309	Mycocerosic acid (C25)	55.8	1.53E-08	0.51
7.66_455.3469m/z	LMFA07070027	3-hydroxyoctadecatrienoylcarnitine	40.3	5.30E-05	0.50
2.80_466.3629n	LMST01030128	6alpha-Hydroxycastasterone	42.3	1.20E-08	0.49
2.84_320.2540m/z	LMSP01080052	Lepadin D	41.4	6.53E-08	0.46
2.79_403.3547m/z	LMST01010335	5alpha-cholestan-3alpha,12alpha,16alpha-triol	37	2.87E-09	0.45
9.24_444.3577n	LMST01010175	3beta-hydroxy-4beta-methyl-5alpha-cholest-7-ene-4alpha-carboxylic acid	41.1	4.04E-11	0.42
12.94_687.5641m/z	LMST01020029	Cholesteryl 11-hydroperoxy-eicosatetraenoate	36.5	1.80E-10	0.37
2.45_485.3809m/z	LMFA01060099	3-oxo-tetradecanoic acid	36.5	9.80E-10	0.37
1.62_330.2618m/z	LMFA02000046	12,13-DiHODE	44.4	8.64E-12	0.34
9.18_802.5913m/z	LMGP03030047	PS(P-38:1)	41	9.03E-10	0.33
6.91_330.3347m/z	LMFA01020220	17-methyl-nonadecanoic acid	49.7	1.32E-12	0.27
8.19_358.3658m/z	LMFA01020019	Behenic acid	48.9	2.53E-17	0.16
1.65_445.2310m/z	LMGP10050002	PA (17:1)	49.8	8.21E-04	0.03

Table S6. AUC value of differential lipids for cancer (BC and RCC) distinction on plasma lipidomics

Name	AUC
11Z-Eicosenal	0.978
6Z-Heneicosen-9-one	0.953
Behenic acid	0.872
17-methyl-nonadecanoic acid	0.815
7Z-Tricosen-11-one	0.804
12,13-DiHODE	0.804
3beta-hydroxy-4beta-methyl-5alpha-cholest-7-ene-4alpha-carboxylic acid	0.795
10Z-Nonadecen-2-one	0.792
Cholesteryl 11-hydroperoxy-eicosatetraenoate	0.785
3-oxo-tetradecanoic acid	0.773
PS(P-38:1)	0.773
5alpha-cholestan-3alpha,12alpha,16alpha-triol	0.766
6alpha-Hydroxycastasterone	0.756
Mycocerosic acid (C25)	0.755
Lepadin D	0.744
Japanic acid	0.738
24:0(17Me)	0.717
6beta-acetoxy-24-methylcholestan-3beta,5alpha,22R,24-tetrol	0.713
22,23-dihydroxycampesterol	0.710
(25S)-5alpha-cholestan-3beta,6alpha,7beta,8beta,15alpha,16beta,26-heptol	0.702

Table S7. Differential metabolites between BC and RCC on plasma metabolomics

**Features**	**Metabolites ID**	**Description**	**Score**	***p*-value**	**Fold Change (BC/RCC)**
5.60_331.2470m/z	HMDB04710	9,10,13-TriHOME	42.3	3.74E-05	4.93
1.08_143.0940n	HMDB30410	L-2-Amino-3-methylenehexanoic acid	39.4	1.74E-05	3.58
5.53_295.2258m/z	HMDB62434	(10E,12Z)-(9S)-9-Hydroperoxyoctadeca-10,12-dienoic acid	40.2	1.34E-03	3.53
1.15_297.1068m/z	HMDB01412	7,8-Dihydropteroic acid	47.3	3.29E-04	3.41
5.30_246.1513m/z	HMDB59773	S-3-oxodecanoyl cysteamine	41.9	2.97E-08	2.91
5.81_269.2104m/z	HMDB41287	16-Hydroxy-10-oxohexadecanoic acid	45.3	1.52E-03	2.58
3.89_203.0476m/z	HMDB29430	Methionine sulfoximine	39.8	1.04E-03	2.01
1.21_276.0948n	HMDB04813	3-Methyluridine	39.6	4.38E-04	1.86
1.55_100.0211m/z	HMDB02287	Homocysteine thiolactone	40.5	1.03E-03	1.78
1.55_146.0266m/z	HMDB01890	Acetylcysteine	45	4.41E-06	1.73
6.60_314.2448n	HMDB29978	Avenoleic acid	39.5	1.47E-03	1.73
3.86_174.0786n	HMDB29739	1H-Indole-3-acetamide	41.1	1.04E-02	1.58
3.78_230.1379m/z	HMDB13127	Hydroxybutyrylcarnitine	45.8	2.90E-02	1.5
1.21_305.1444m/z	HMDB02089	N-Ribosylhistidine	50	2.98E-03	1.45
4.23_283.0836n	HMDB60950	fluvoxamino acid	53.9	4.73E-03	1.32
3.72_262.1641m/z	HMDB62555	hydroxyisovaleroyl carnitine	39.7	1.43E-03	1.23
9.46_448.3618m/z	HMDB29888	Sorbitan stearate	38.1	3.32E-02	1.01
0.78_107.0304m/z	HMDB29634	2-Methylbenzenethiol	36.8	2.48E-03	0.98
1.01_189.0862m/z	HMDB29049	Serinyl-Threonine	45.1	1.55E-04	0.87
1.12_263.1227m/z	HMDB02104	L-Glutamic gamma-semialdehyde	45.5	4.96E-02	0.8
5.93_208.1073m/z	HMDB62444	3-(Hydroxymethyl)nitrosoamino]propyl)-3-pyridinemethanol	40.7	4.39E-07	0.68
4.85_372.2654n	HMDB31126	3,4-Dimethyl-5-pentyl-2-furanundecanoic acid	53.1	2.61E-05	0.64
4.33_184.0366n	HMDB13198	4-O-Methylgallic acid	38.7	4.97E-03	0.62
5.90_307.2258m/z	HMDB38685	Muricatacin	48.1	5.22E-04	0.51

Table S8. AUC value of differential metabolites for BC and RCC distinction on plasma metabolomics

Name	AUC
16-Hydroxy-10-oxohexadecanoic acid	0.812
7,8-Dihydropteroic acid	0.805
9,10,13-TriHOME	0.785
S-3-oxodecanoyl cysteamine	0.759
(10E,12Z)-(9S)-9-Hydroperoxyoctadeca-10,12-dienoic acid	0.755
Avenoleic acid	0.755
3,4-Dimethyl-5-pentyl-2-furanundecanoic acid	0.730
L-2-Amino-3-methylenehexanoic acid	0.720
Muricatacin	0.708

Table S9. Differential lipids between BC and RCC on plasma lipidomics

Features	Metabolites ID	Description	Score	*p*-value	Fold Change (BC/RCC)
9.08_818.5857m/z	LMGP04010600	PG(38:3)	51.6	3.97E-02	1.79
5.93_506.3210m/z	LMGP01050127	PC(17:2)	45.7	4.70E-03	1.59
8.33_298.3086m/z	LMFA12000291	9S,10R-Epoxy-6Z-nonadecene	46.2	1.15E-02	1.28
2.34_447.3442m/z	LMST01010246	Trihydroxycoprostanoic acid	49.2	3.14E-02	0.92
5.52_468.3186n	LMST03020603	2beta-methoxy-1alpha,25-dihydroxyvitamin D3	43.2	1.41E-02	0.91
2.95_490.3630n	LMST01031065	6beta-acetoxy-24-methylcholestan-3beta,5alpha,22R,24-tetrol	39.3	3.37E-02	0.88
4.45_432.3578n	LMST01031125	22,23-dihydroxycampesterol	54.1	2.20E-02	0.86
11.44_666.6353m/z	LMSP02030002	Cer(t18:0/24:0(2OH))	39.1	3.46E-02	0.83
4.02_519.4015m/z	LMST03020631	(20R)-24-Hydroxygeminivitamin D3	41.1	3.56E-02	0.83
11.83_640.5833m/z	LMGL02010043	DG(36:1)	36.1	3.10E-02	0.82
2.36_482.3813m/z	LMST03020676	24,24-Difluoro-25-hydroxy-26,27-dimethylvitamin D3	39.4	2.77E-02	0.82
2.75_467.3703m/z	LMPR01070189	4'-Apo-3,4-didehydrolycopene/ (4-Apo-3',4'-didehydrolycopene)	38	4.09E-02	0.78
4.51_538.4198n	LMFA03050002	12S-HHTrE	43.2	1.12E-02	0.78
3.23_320.2539m/z	LMSP01080002	4E,14Z-Sphingadiene	40.3	1.92E-04	0.6
8.39_826.5905m/z	LMGP03030046	PS(P-38:0)	43.9	7.74E-07	0.45
2.18_367.2823m/z	LMFA01050426	Tetrapedic acid A	47.8	1.89E-04	0.34
6.22_650.4351m/z	LMGP20010008	PC(16:0/9:0(CHO))	40.3	5.55E-03	0.3

Table S10. AUC value of differential lipids for BC and RCC distinction on plasma lipidomics

Name	AUC
PS(P-38:0)	0.792
4E,14Z-Sphingadiene	0.708
Tetrapedic acid A	0.704

Table S11 Eight common differential metabolites in BC (64 samples), RCC (74 samples) and control (141 samples) group.

Label	BC vs Control		RCC vs Control		BC vs RCC	
	P.value	FC[(a)]	P.value	FC[(b)]	P.value	FC[(c)]
16-Hydroxy-10-oxohexadecanoic acid	3.14E-28	0.15	3.52E-32	0.07	6.28E-06	2.01
9,10,13-TriHOME	1.04E-26	0.18	2.57E-31	0.07	3.04E-06	2.52
(10E,12Z)-(9S)-9-Hydroperoxyoctadeca-10,12-dienoic acid	1.04E-26	0.21	2.57E-31	0.10	2.11E-05	2.22
Avenoleic acid	8.76E-05	0.27	6.41E-28	0.19	4.23E-05	1.47
Methionine sulfoximine	2.07E-04	0.32	6.32E-14	0.12	1.64E-03	2.72
Homocysteine thiolactone	1.47E-03	0.43	5.75E-08	0.19	1.51E-02	2.30
Acetylcysteine	1.96E-02	0.47	1.14E-06	0.21	4.40E-04	2.18
9S,10R-Epoxy-6Z-nonadecene	4.47E-25	3.24	3.79E-16	2.36	1.62E-04	1.38

a. FC[(a)]: Fold Change(BC/Control)

b. FC[(b)]: Fold Change(RCC/Control)

c. FC[(c)]: Fold Change(BC/RCC)

Table S12. The advantages and limitations of NMR spectroscopy and MS spectrometry as an analytical tool for metabolomics research adapted from ref.(Emwas AH, et al. *Methods Mol Biol*. 2015;1277:161-93.**)**

Technology	Advantages	Limitation
NMR	good reproducibility, minimal sample preparation requirements, non-destructive detection and precise quantification	low sensitivity；Not relevant for targeted analysis；expensive
LC-MS	high sensitivity；high Resolution；enables the measurement of hundreds of individual species within a single sample.	the lack of database；MS signal intensity of any compound is affected by the type of sample preparation used and its molecular environment.
GC-MS	high Resolution, high selectivity, and sound database	Sample processing is cumbersome and needs to be derivatized

Supplementary Figures

Fig. S1 Assessment of QC samples. **a.** Trend plot showing the variation of t [1] over all QC Samples on plasma metabolomics. X axis numbers represented sample number, Y axis was arbitrary (3 s.d.); **b.** Trend plot showing the variation of t [1] over all QC Samples on plasma lipidomics. **c.** PC1 versus PC2 of test samples and QC on plasma metabolomics. **d.** PC1 versus PC2 of test samples and QC on plasma lipidomics.

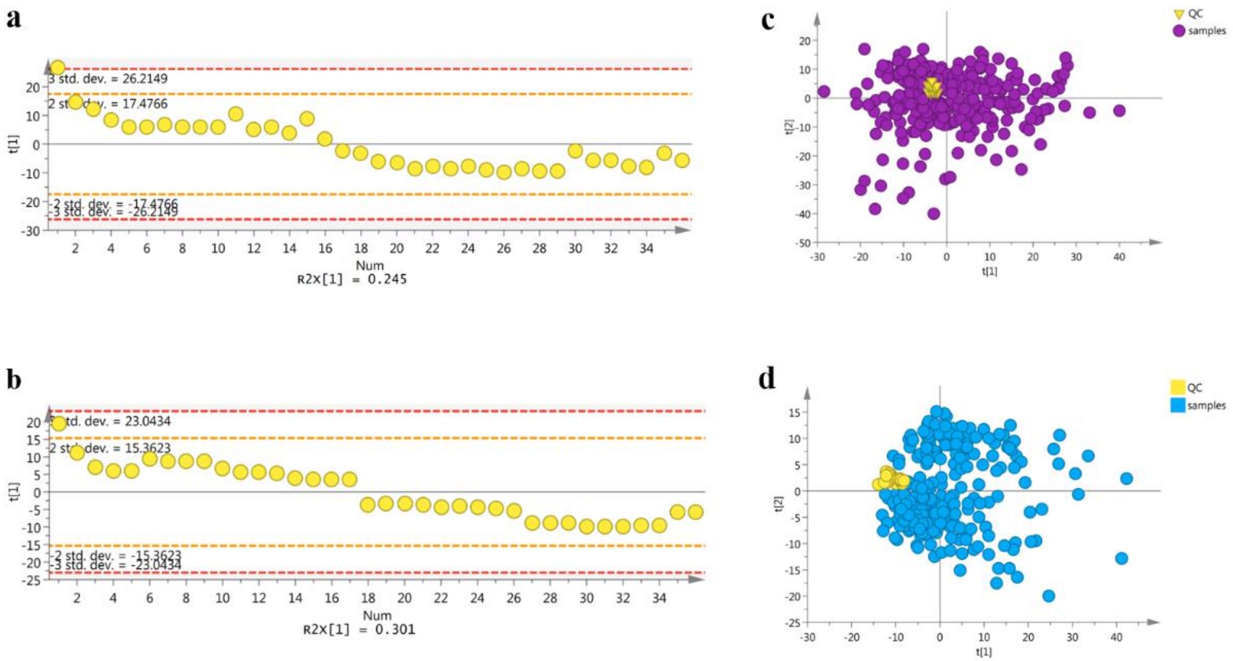

Fig. S2 Analysis of metabolic profiling variation of 95 cancer (BC and RCC) samples and 95 control samples. **a.** Score plot of unsupervised PCA overview of plasma metabolic profiling between cancer and control. **b.** 100 permutation tests of the OPLS-DA model based on cancer and control plasma metabolomics. **c.** Top five shifted metabolic pathways in cancer (BC and RCC) compared with control. **d.** ROC plot with discovery group for distinction of cancer and control based on 9,10,13-TriHOME, 12,13-DHOME and linolenelaidic acid. **e.** ROC plot with external validation group for distinction of 43 cancer samples and 46 control samples based on 9,10,13-TriHOME, 12,13-DHOME and linolenelaidic acid.

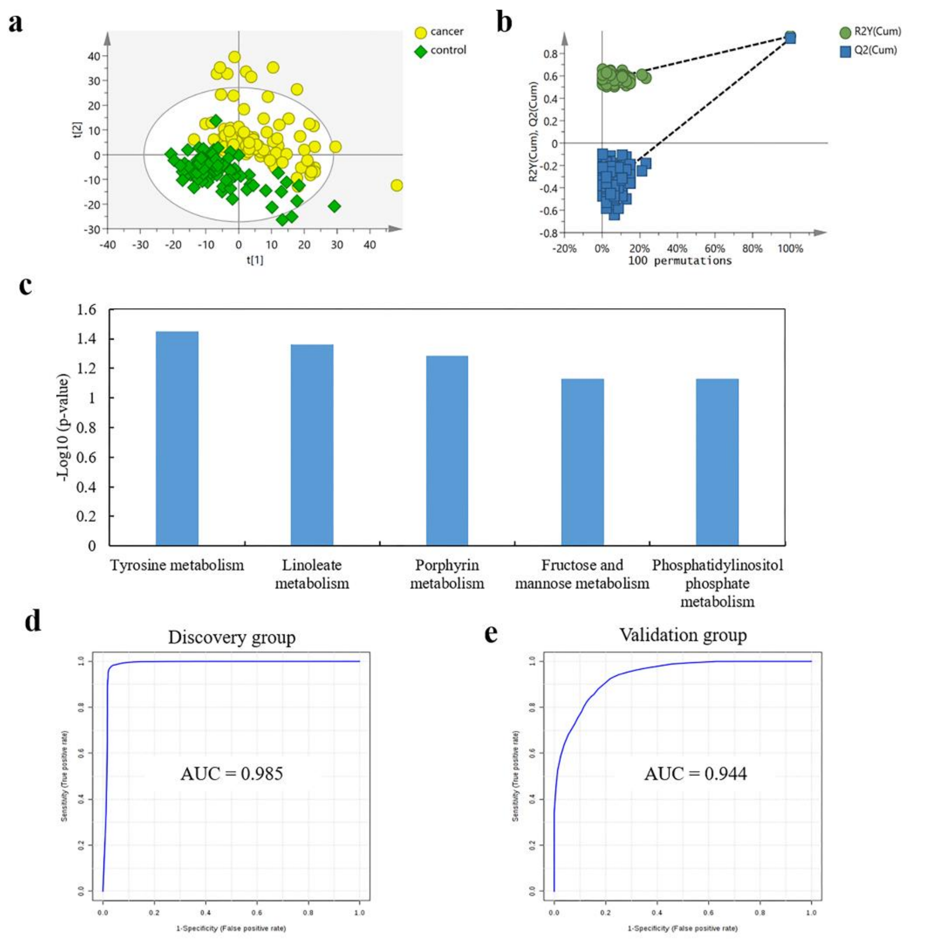

Fig. S3 Analysis of lipidomic profiling variation of 95 cancer (BC and RCC) samples and 95 control samples. **a.** Score plot of unsupervised PCA overview of plasma lipidomic profiling between cancer and control. **b.** 100 permutation tests of the OPLS-DA model based on cancer and control plasma lipidomics. **c.** Top five shifted lipidomic pathways in cancer (BC and RCC) compared with control. **d.** ROC plot with discovery group for distinction of cancer and control based on 11Z-Eicosenal, 6Z-Heneicosen-9-one, behenic acid and 7Z-Tricosen-11-one. **e.** ROC plot with external validation group for distinction of 43 cancer samples and 46 control samples based on 11Z-Eicosenal, 6Z-Heneicosen-9-one, behenic acid and 7Z-Tricosen-11-one.

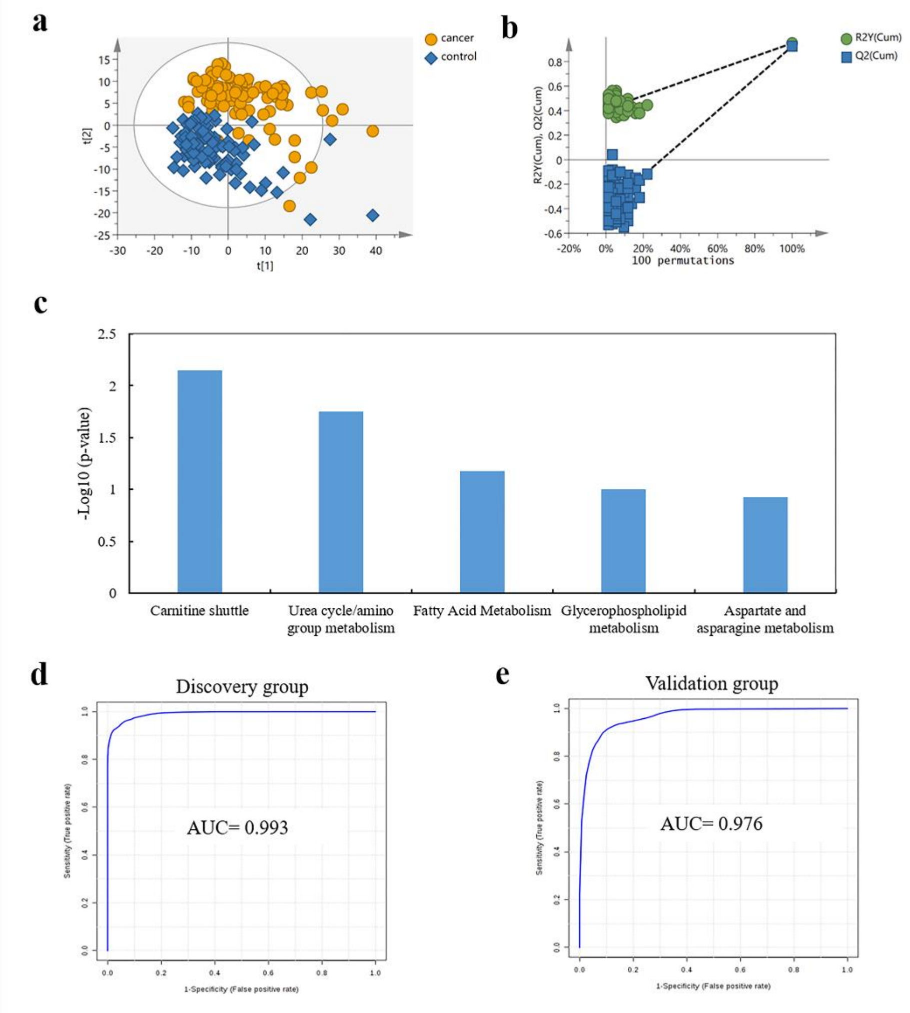

Fig. S4 Analysis of metabolic profiling variation of 42 BC samples and 53 RCC samples. **a.** Score plot of unsupervised PCA overview of plasma metabolic profiling between BC and RCC. **b.** 100 permutation tests of the OPLS-DA model based on BC and RCC plasma metabolomics. **c.** Top five shifted metabolic pathways in BC and RCC. **d.** ROC plot with discovery group for distinction of BC and RCC based on 7,8-Dihydropteroic acid, Avenoleic acid and 3,4-Dimethyl-5-pentyl-2-furanundecanoic acid. **e.** ROC plot with external validation group for distinction of 22 BC samples and 21 RCC samples based on 7,8-Dihydropteroic acid, Avenoleic acid and 3,4-Dimethyl-5-pentyl-2-furanundecanoic acid.

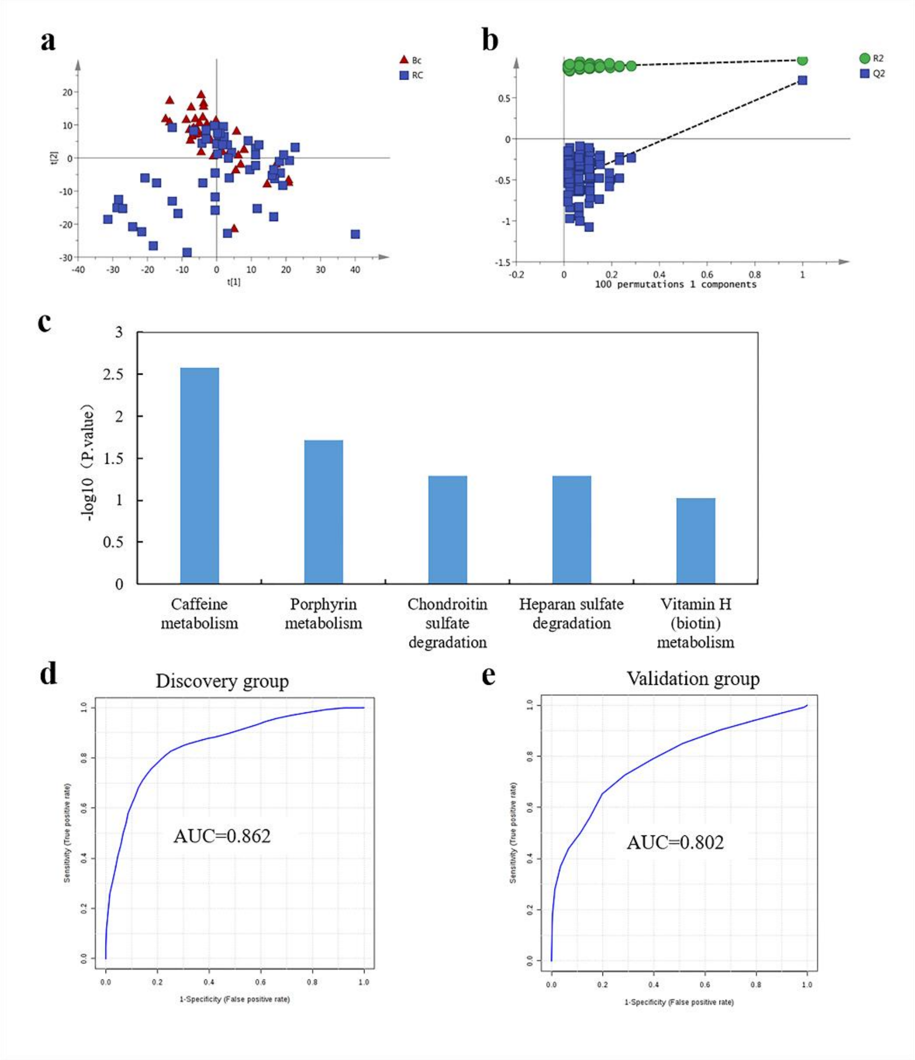

Fig. S5 Analysis of lipidomic profiling variation of 42 BC samples and 53 RCC samples. **a.** Score plot of unsupervised PCA overview of plasma lipidomic profiling between BC and RCC. **b.** 100 permutation tests of the OPLS-DA model based on BC and RCC plasma lipidomics. **c.** Top five shifted lipidomic pathways in BC and RCC. **d.** ROC plot with discovery group for distinction of BC and RCC based on PS(P-38:0), 4E,14Z-Sphingadiene and Tetrapedic acid A. **e.** ROC plot with external validation group for distinction of 22 BC samples and 21 RCC samples based on PS(P-38:0), 4E,14Z-Sphingadiene and Tetrapedic acid A.

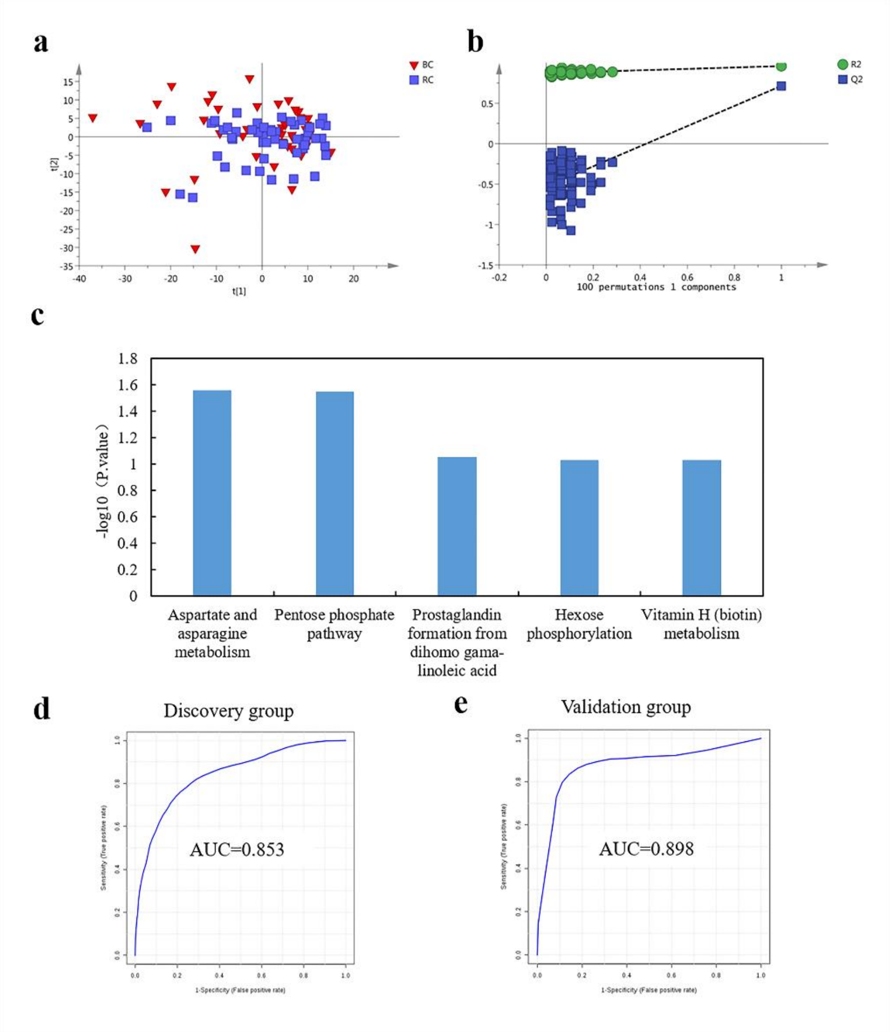

Fig. S6 Typical base peak chromatograms from representative serum samples (a) Typical base peak chromatograms of plasma metabolomics. Top：control sample; Middle: Renal cell carcinoma sample; Down: bladder cancer sample. (b) Typical base peak chromatograms of plasma lipidomics. Top：bladder cancer sample; Middle: control sample; Down: Renal cell carcinoma sample.

(a) Typical base peak chromatograms of plasma metabolomics. Top：control sample; Middle: Renal cell carcinoma sample; Down: bladder cancer sample.

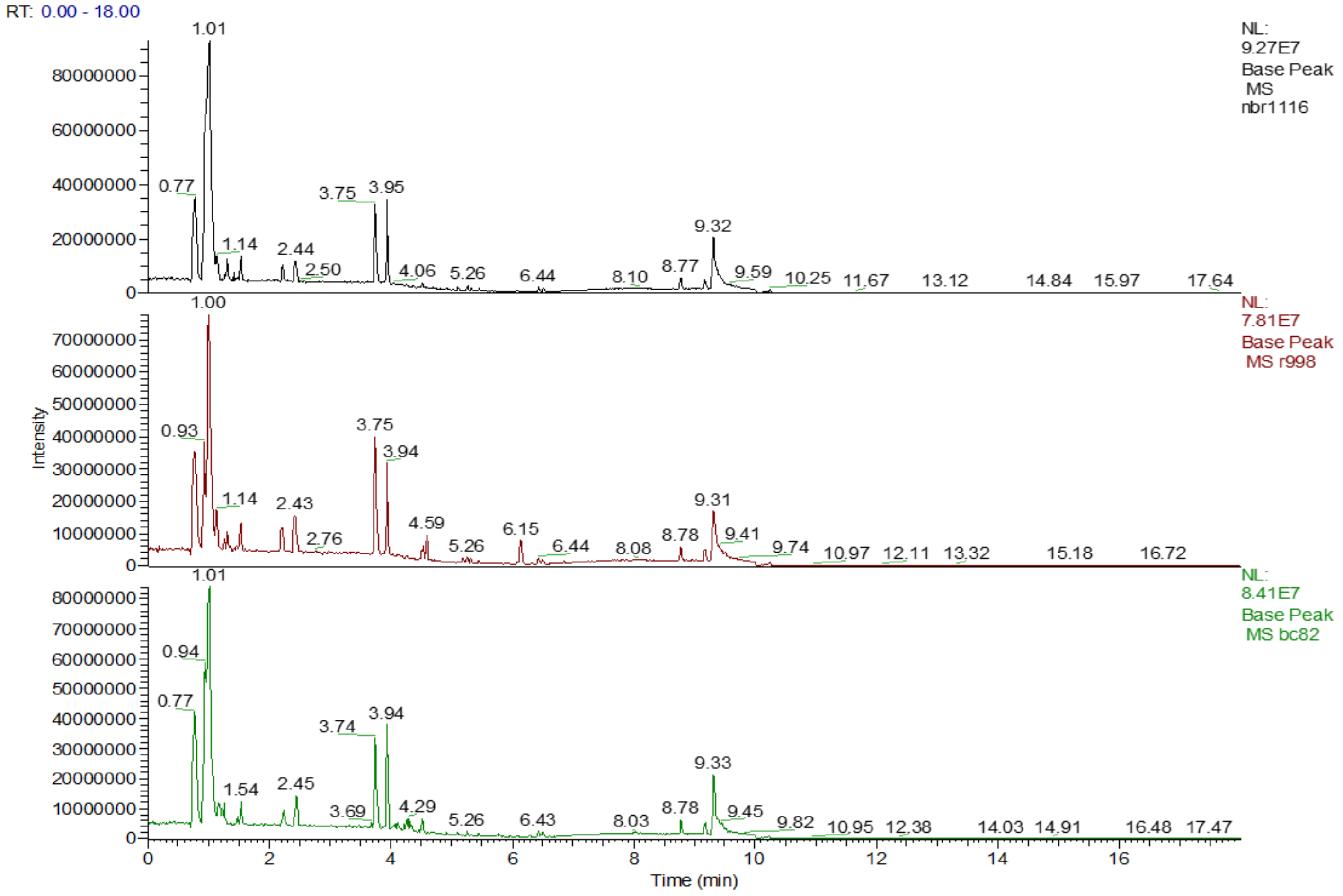

(b) Typical base peak chromatograms of plasma lipidomics. Top：bladder cancer sample; Middle: control sample; Down: Renal cell carcinoma sample.

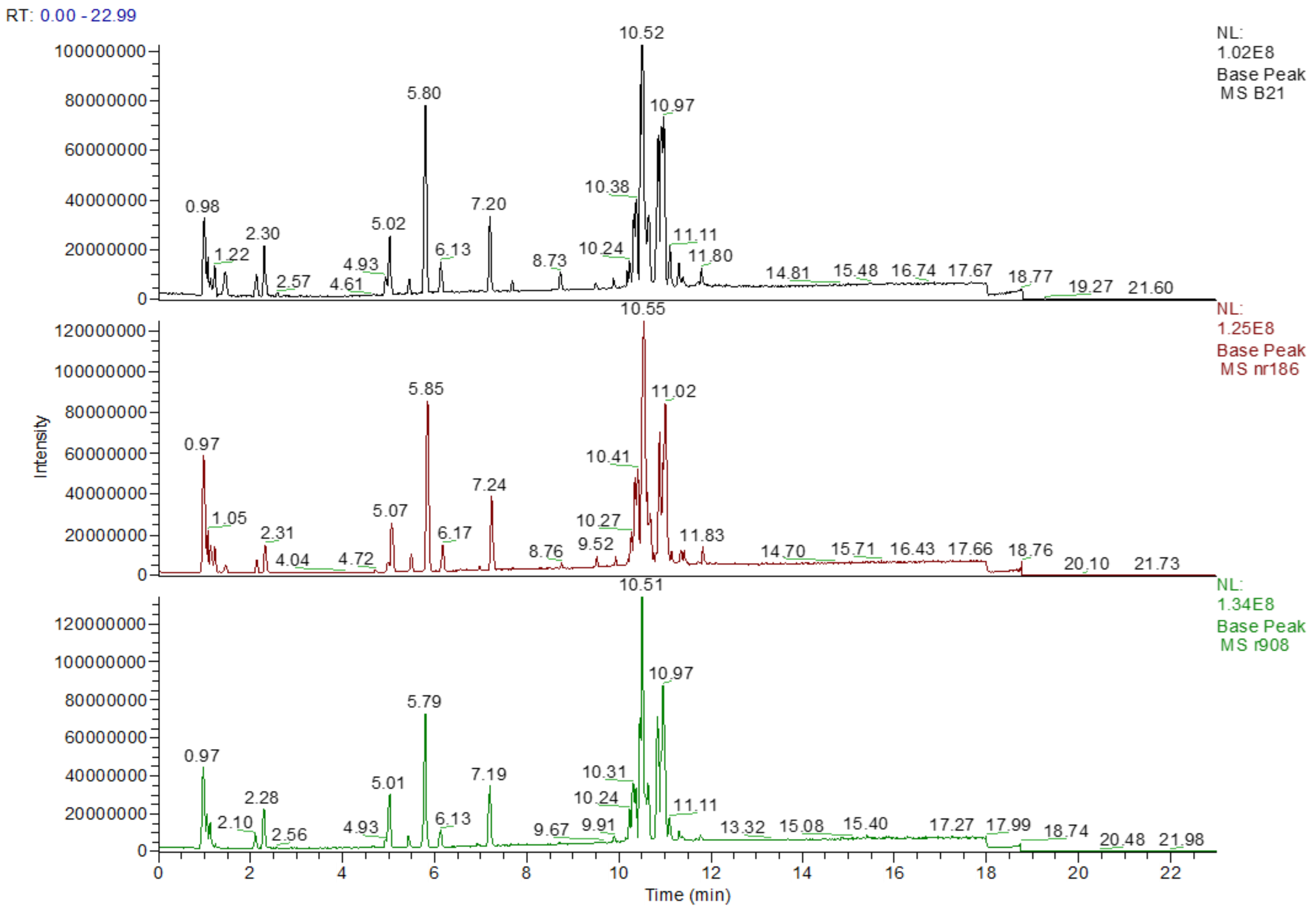

Fig S7 MS2 spectra of 5 metabolites identified in standard compounds (STD) and in QC sample. (a) hypoxanthine, (b) homocysteine thiolactone, (c) 4-Ethylphenol, (d) L-octanoylcarnitine, (e) acetylcysteine

a. Hypoxanthine m/z: 137.05 score: 41.1

(1) STD

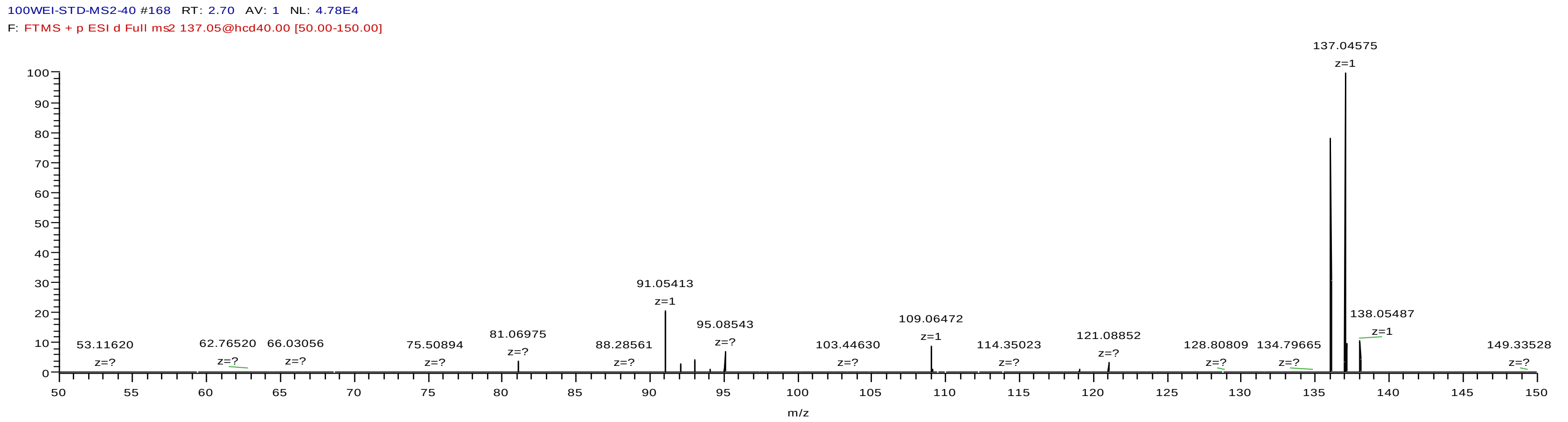

(2) QC

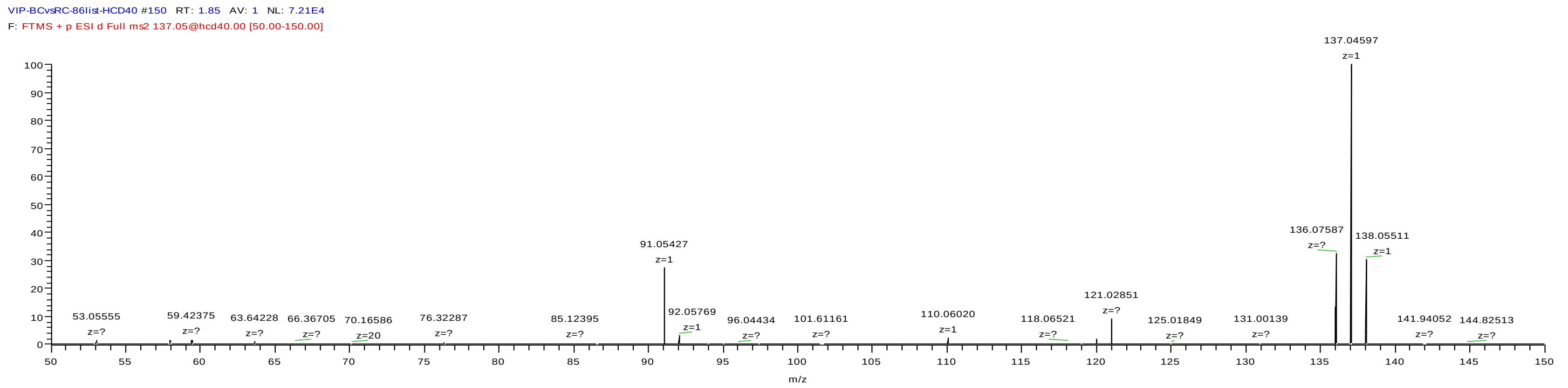

b. Homocysteine thiolactone m/z: 100.02 score: 40.5

(1) STD

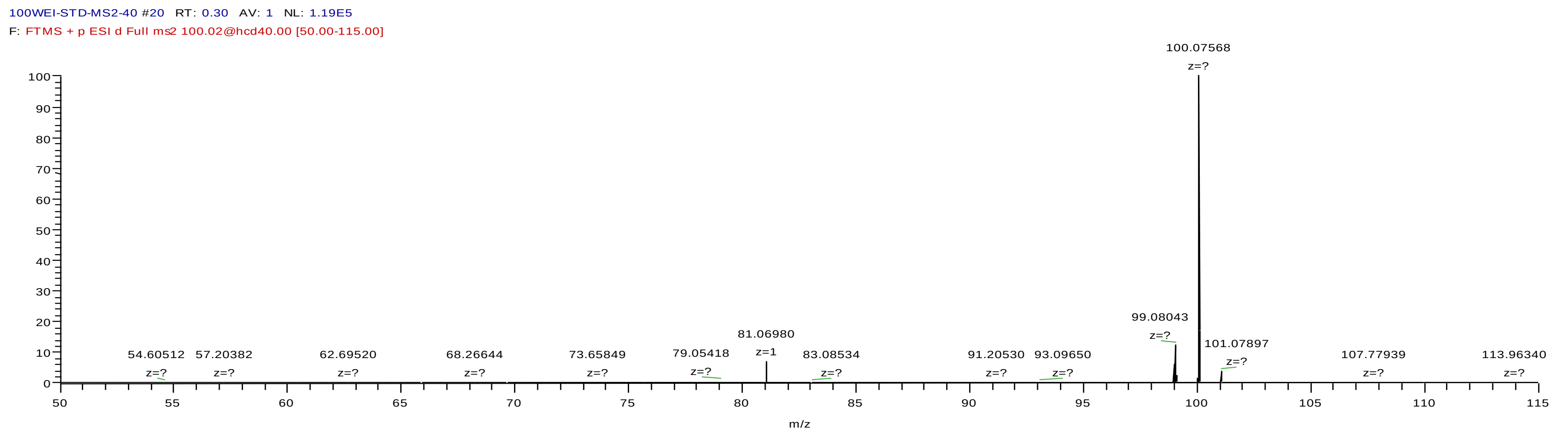

(2) QC

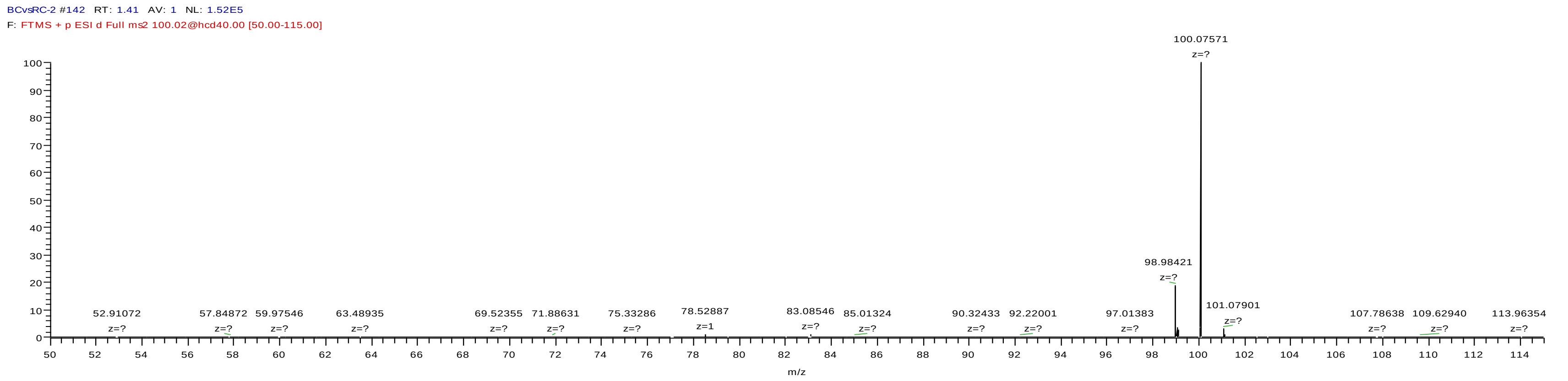

c. 4-Ethylphenol m/z: 105.07 score: 39

(1) STD

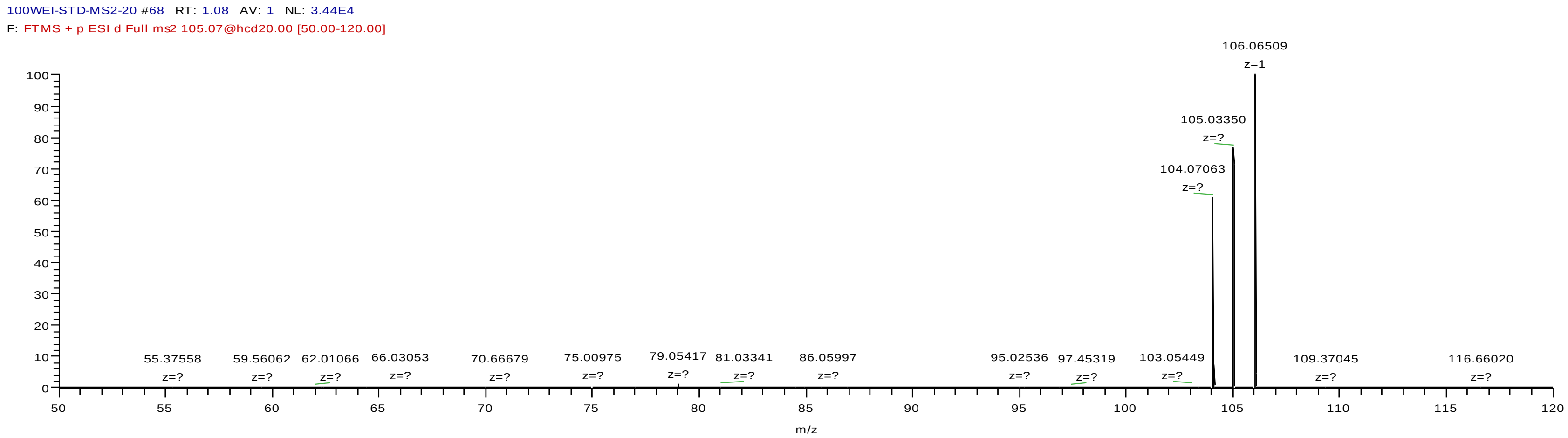

(2) QC

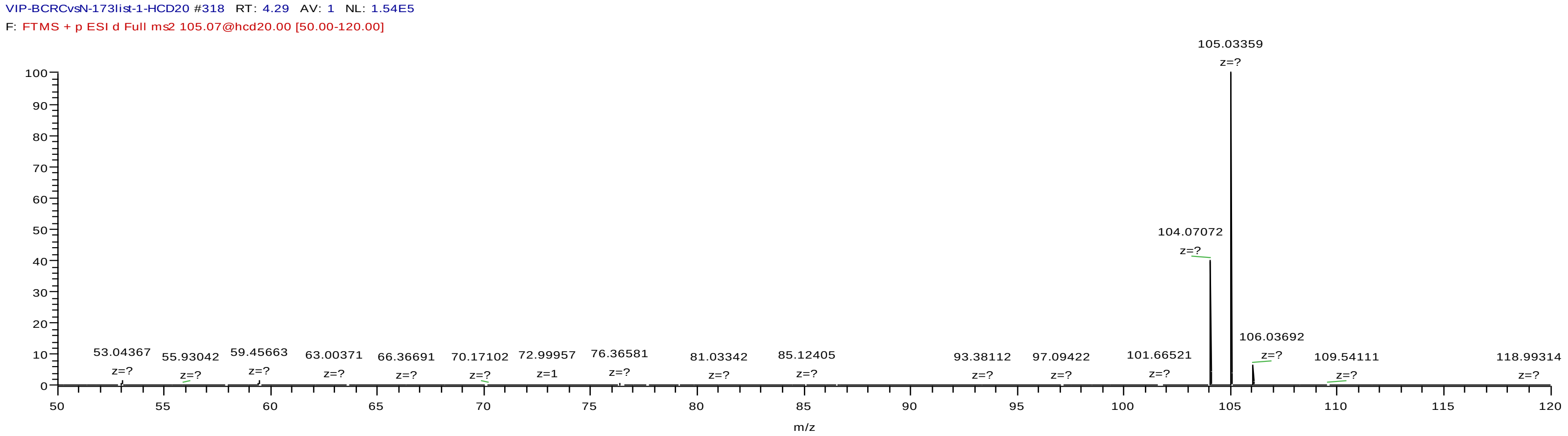

d. L-Octanoylcarnitine m/z: 288.22 score: 51.7

(1) STD

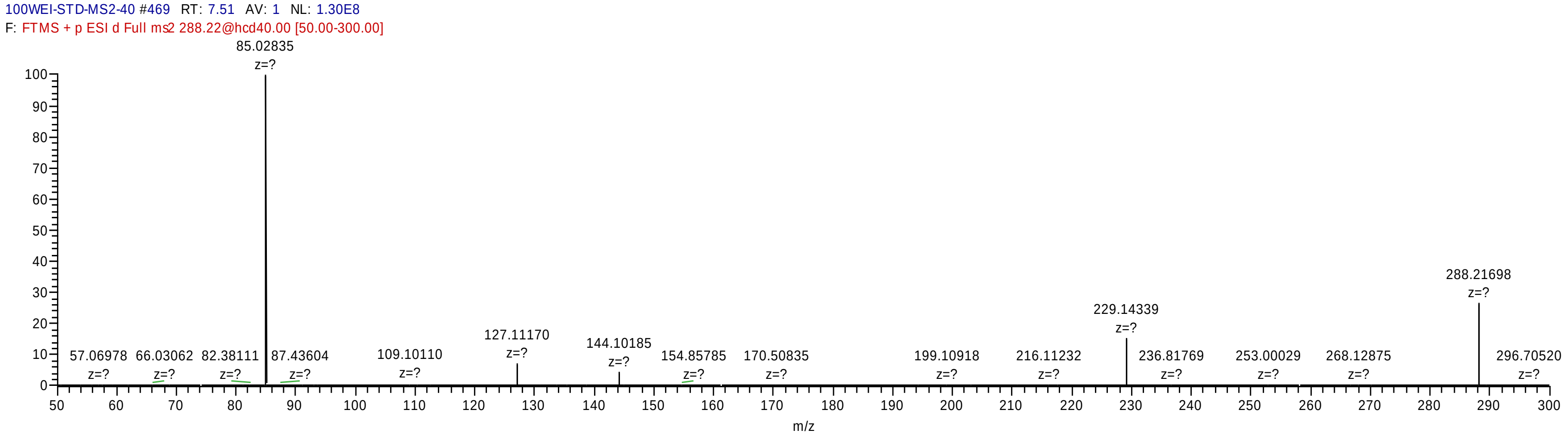

(2) QC

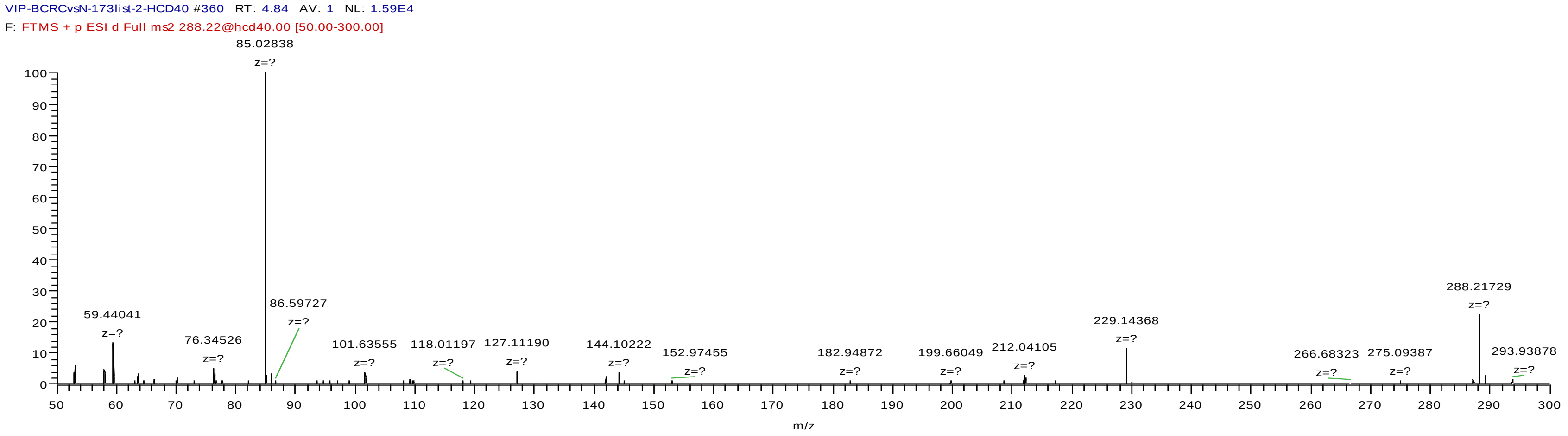

e. Acetylcysteine m/z 146.03 score: 45

(1) STD

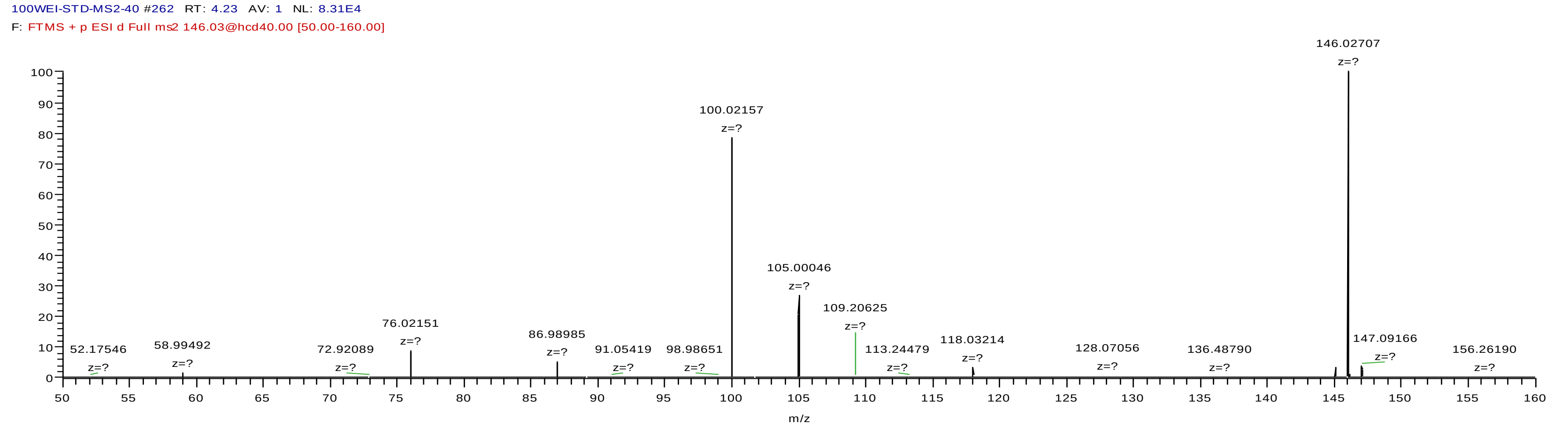

(2) QC

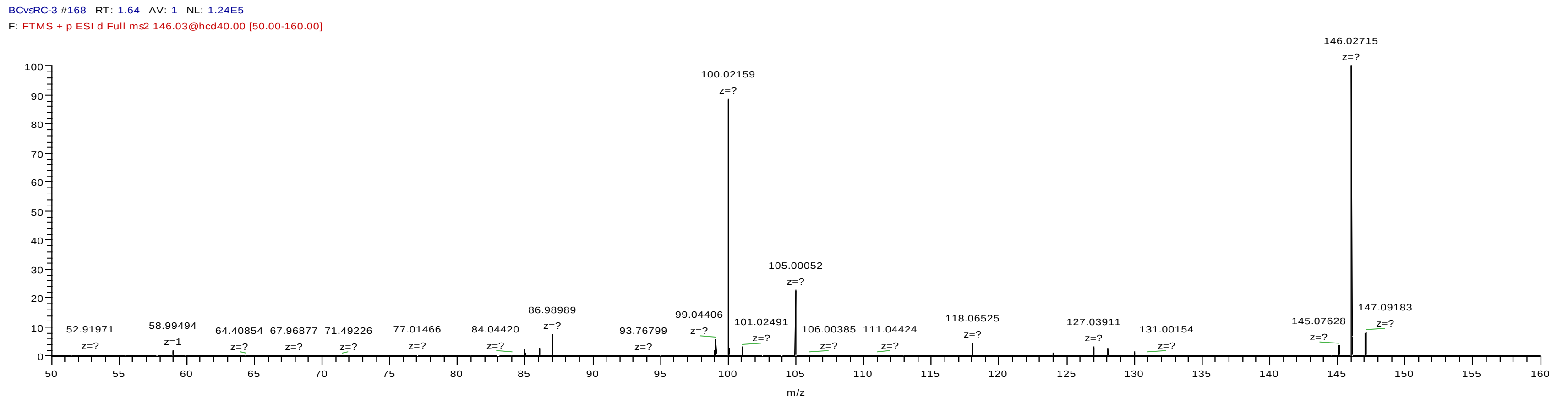

Supplement: Supplementary file 1 [file Data_Sheet_1.PDF]
